# A Plant Virus Glycoprotein Induces Autophagy by Activating the Toll7 Immune Pathway in Its Insect Vector

**DOI:** 10.1111/mpp.70259

**Published:** 2026-04-12

**Authors:** Yu‐Juan He, Yu‐Hua Qi, Can Wang, Gao‐Yang Jiao, Zhuang‐Xin Ye, Lin Lin, Qian‐Zhuo Mao, Gang Lu, Xiao‐Wei Wang, Chuan‐Xi Zhang, Jian‐Ping Chen, Jun‐Min Li

**Affiliations:** ^1^ State Key Laboratory for Quality and Safety of Agro‐Products, Key Laboratory of Biotechnology in Plant Protection of MARA, Zhejiang Key Laboratory of Green Plant Protection, Institute of Plant Virology Ningbo University Ningbo China

**Keywords:** autophagy, glycoprotein, *Laodelphax striatellus*, PI3K‐Akt–mTOR pathway, rice stripe virus, Toll7

## Abstract

The Toll7 pathway is crucial in defending against diverse pathogenic microorganisms, including viruses. This study reveals that a plant virus (rice stripe virus, RSV) infection activates Toll7‐mediated antiviral response in the insect vector *Laodelphax striatellus* . We identified a specific interaction between the TIR domain of Toll7 and RSV glycoprotein (Gc). Furthermore, *Toll7* silencing significantly enhanced RSV replication and acquisition, suggesting its antiviral role in *L. striatellus* . Transcriptomic analysis indicated that Toll7 negatively regulates the PI3K‐Akt–mTOR pathway, a key autophagy regulator. *Toll7* knockdown upregulated the expression of PI3K, Akt and mTOR; simultaneously, autophagy‐related genes (*Atg3*, *Atg5*, *Atg8*, *Atg9*, *Torc1* and *ULK1*) were downregulated and autophagy inhibitor Sqstm1 was upregulated. Conversely, silencing *PI3K*, *Akt* or *mTOR* suppressed RSV replication, highlighting the essential role of this pathway in viral persistence within its vector. These findings demonstrate that Toll7‐mediated inhibition of the PI3K‐Akt–mTOR pathway activates autophagy, restricting RSV replication in *L. striatellus* . This study uncovers a conserved Toll7‐dependent antiviral mechanism modulating autophagy to inhibit viral infection, offering new insights into the co‐evolutionary dynamics between plant viruses and insect vectors.

## Graphical abstract

The induction of autophagy in *Laodelphax striatellus* by Rice stripe virus (RSV) glycoprotein through the activation of the Toll7 immune pathway.

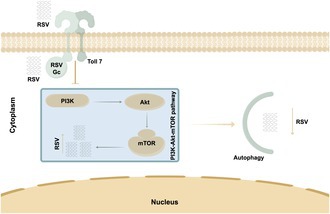

## 1 Introduction

In the innate immunity of metazoans, Toll receptors, as key pattern recognition receptors (PRRs), play a vital role in detecting and eliminating a wide range of pathogens, including bacteria, fungi, viruses and protozoa (Janeway Jr and Medzhitov 2002). Pathogens are recognized directly or indirectly by Toll receptors through pathogen‐associated molecular patterns (PAMPs), which in turn initiate multiple distinct cellular signalling cascade pathways (Barton 2007; Hemmi et al. 2000; Takeda and Akira 2003). The Toll‐Dorsal signalling pathway is a well‐established signalling cascade, primarily involved in the defence against infections caused by gram‐positive bacteria and fungi (Valanne et al. 2011; Belvin and Anderson 1996). Notably, lipopolysaccharide or peptidoglycan in these bacterial cell walls serves as PAMPs, which subsequently trigger a proteolytic cascade leading to the activation of Spätzlea, the cytokine that binds to Toll receptors, facilitating the recruitment of MyD88, Tube and Pelle to form the receptor‐proximal oligomeric complex, thereby initiating an NF‐κB‐dependent transcriptional programme for antimicrobial defence (Horng and Medzhitov 2001; Towb et al. 2001; Sun et al. 2002; Barton 2007). Unlike bacteria or fungi, viruses activate distinct downstream signalling cascades through different Toll receptors to induce antiviral responses in host cells either directly or indirectly. In *Drosophila*, the canonical Toll‐Dorsal signalling pathway is known to be involved in mediating resistance to several RNA viruses, including Drosophila X virus (DXV), Drosophila C virus (DCV), cricket paralysis virus, flock house virus and norovirus (Zambon et al. 2005; Ferreira et al. 2014). In mosquitoes, the canonical Toll‐Dorsal signalling pathway also plays a role in the anti‐dengue viruses defences (Xi et al. 2008). In studies on *Litopenaeus vannamei* , the white spot syndrome virus directly binds to the Toll4 receptor, activating the canonical Toll‐Dorsal signalling pathway (Li et al. 2018). Our previous studies also elucidated that Toll‐Dorsal‐ZN708 mediates the homeostasis maintenance of rice stripe virus (RSV) in *Laodelphax striatellus* (He et al. 2024, 2021).

In contrast, upon viral activation of the Toll7 receptor, distinct signalling cascades are initiated that differ fundamentally from the canonical Toll‐Dorsal signalling pathway. An experiment in mice demonstrated that Toll‐like receptor 7 (TLR7) and its adaptor protein MyD88 are critical for the host response to vesicular stomatitis virus (VSV) and influenza virus infection. The underlying mechanism involves the binding of viral single‐stranded RNA (ssRNA) to the leucine‐rich repeat (LRR) domain of TLR7, inducing a conformational change that promotes receptor dimerization. This, in turn, facilitates the recruitment of MyD88, triggering intracellular signalling cascades that culminate in the production of interferons (type I IFNs) and pro‐inflammatory cytokines (Lund et al. 2004). Interestingly, studies in *Drosophila* have shown that the glycoprotein of VSV interacts with the Toll7 receptor, leading to the induction of autophagy (Nakamoto et al. 2012). The induction of autophagy triggered by VSV is achieved through the inhibition of TOR in the phosphoinositide‐3‐kinase–protein kinase B/Akt (PI3K‐Akt) signalling pathway (Cherry 2009). Similarly, a study in both *Drosophila* and mammals demonstrated that the Toll7 receptor specifically binds to and recognizes Rift Valley fever virus (RVFV), leading to the activation of TNF receptor‐associated factor 6 (TRAF6). TRAF6, in turn, initiates antiviral autophagy by reducing the activation of Akt (Moy et al. 2014). It is worth noting that when RVFV and VSV simultaneously infect mammalian cells, only RVFV induces autophagy (Moy et al. 2014). The results demonstrated that VSV, after binding to Toll7 in cells from different species (*Drosophila* and mammalian cells), induces distinct antiviral mechanisms (Nakamoto et al. 2012; Cherry 2009; Moy et al. 2014). The potential cause for this discrepancy may lie in the fact that, although both the *Drosophila* Toll7 receptor and mammalian TLR7 belong to the Toll/TLR superfamily, they have undergone distinct evolutionary trajectories and functional divergence (Leulier and Lemaitre 2008). Beyond the study of animal viruses, southern rice black‐streaked dwarf virus (SRBSDV) activates insect Toll7 but inhibits antiviral defence by degrading MyD88 via its P7‐1 protein and RING E3 ligase (Jia et al. 2024). In other insect vectors, it remains to be explored whether plant viruses trigger additional signalling cascades upon activating Toll7.

The PI3K‐Akt pathway has been extensively recognized as a crucial signalling axis involved in regulating cell growth, proliferation and survival (Ersahin et al. 2015; Cho et al. 2009; Xi et al. 2015). Generally, upon receiving external stimuli (e.g., growth factors), class I PI3K is activated. Activated PI3K phosphorylates PIP2, converting it into PIP3, a reaction that can be reversed by the phosphatase PTEN (Czech 2000). This molecule serves as a lipid second messenger and can regulate the phosphorylation of various kinases, including Akt. Akt is activated through phosphorylation at Thr308 and Ser473 (Hart and Vogt 2011; Bayascas and Alessi 2005). Phosphorylated Akt is subsequently associated with cell survival, proliferation, migration, differentiation and apoptosis (Alessi et al. 1996; Manning and Cantley 2007; Yao and Cooper 1995; Datta et al. 1999). As a key downstream regulatory molecule of the PI3K‐Akt signalling pathway, mTOR is activated by this pathway to promote cell survival and proliferation (Saxton and Sabatini 2017). Moreover, activated mTOR not only facilitates cell proliferation by enhancing anabolic processes but also suppresses catabolic processes, such as autophagy, under metabolic stress conditions, thereby driving necrosis or non‐apoptotic cell death (Wu et al. 2009). Coincidentally, as discussed earlier, VSV induces autophagy by reducing Akt levels in the PI3K‐Akt signalling pathway, thereby inhibiting TOR expression (Nakamoto et al. 2012; Cherry 2009; Moy et al. 2014). The induction of autophagy is tightly associated with the inhibition of the PI3K‐Akt–mTOR pathway under stress conditions (Xu et al. 2020; Heras‐Sandoval et al. 2014). Moreover, animal viruses have been shown to induce autophagy via this mechanism (Lin et al. 2020; Cherry 2009; Chang et al. 2017). However, it remains to be investigated whether a similar mechanism exists in vector insects to counteract the persistent infection of plant arboviruses within vector insects.

RSV is transmitted in rice by *L. striatellus* through a persistent‐propagative manner (Falk and Tsai 1998; Toriyama 1986). Upon feeding, viral particles penetrate the midgut epithelium, replicate systemically and migrate via haemolymph to salivary glands for horizontal spread. Notably, RSV colonizes vector reproductive organs (ovaries/testes), enabling transovarial transmission (Hogenhout et al. 2008; Xu et al. 2021). The development of *L. striatellus* requires ~14 days at 25°C with temperature‐dependent variation. Nymphs (particularly second instar) demonstrate superior tenuivirus transmission efficiency compared to adults, and a critical 4‐day latency precedes viral transmissibility (Xiao et al. 2023). The genome of RSV comprises four ssRNA segments (RNA1–4). RNA1 (negative‐sense) encodes the 337 kDa RNA‐dependent RNA polymerase (RdRp) (Toriyama et al. 1994). Ambisense RNA2–4 each encode dual proteins: RNA2 produces silencing suppressor NS2 and glycoprotein NSvc2 via complementary strands (Takahashi et al. 1993; Yao et al. 2014; Zheng et al. 2015). RNA3 generates suppressor NS3 and nucleocapsid protein (NP) (Wu et al. 2014). RNA4 encodes virulence factor NS4 and movement protein (MP) through bidirectional transcription (Hayano et al. 1990). *L. striatellus* employs multimodal antiviral defences against RSV. RNAi immunity initiates through vector‐derived siRNAs accumulation during RSV or RBSDV coinfection (Li et al. 2013). While JNK activation enhances viral replication, Atg8 silencing disrupts this pathway (Wang et al. 2017). RSV subverts haemolymph immunity by suppressing prophenoloxidase activity and hijacking *importin α*‐NP interactions for cellular entry (Chen et al. 2020; Zhao et al. 2022; Yu et al. 2021). JAK–STAT signalling paradoxically sustains persistent infection through apoptosis induction (Zhang et al. 2023). Our previous findings elucidated a new Toll pathway antiviral mechanism: Dorsal transcription factor activates LsZN708‐mediated defences, whereas viral NS4 subverts immunity by blocking MSK2‐dependent Dorsal phosphorylation, effectively neutralizing Toll‐mediated resistance (He et al. 2024). However, whether there are additional Toll‐mediated antiviral immune pathways in *L. striatellus* remains to be explored.

In our current study, we revealed that the TIR domain of Toll7 protein interacts with the glycoprotein (Gc) protein of RSV, leading to the inhibition of the PI3K‐Akt signalling pathway in *L. striatellus* . This subsequently suppresses the expression of the mTOR protein complex, thereby activating autophagic responses in *L. striatellus* to exert antiviral immunity. Our results reveal the molecular mechanism by which Toll7 and the PI3K‐Akt–mTOR pathway contribute to maintaining the balance of arboviruses within insect vectors, which facilitates viral transmission in the field.

## 2 Results

### 2.1 Active Response of Toll7 Pathway During RSV Infection

**FIGURE 1:**
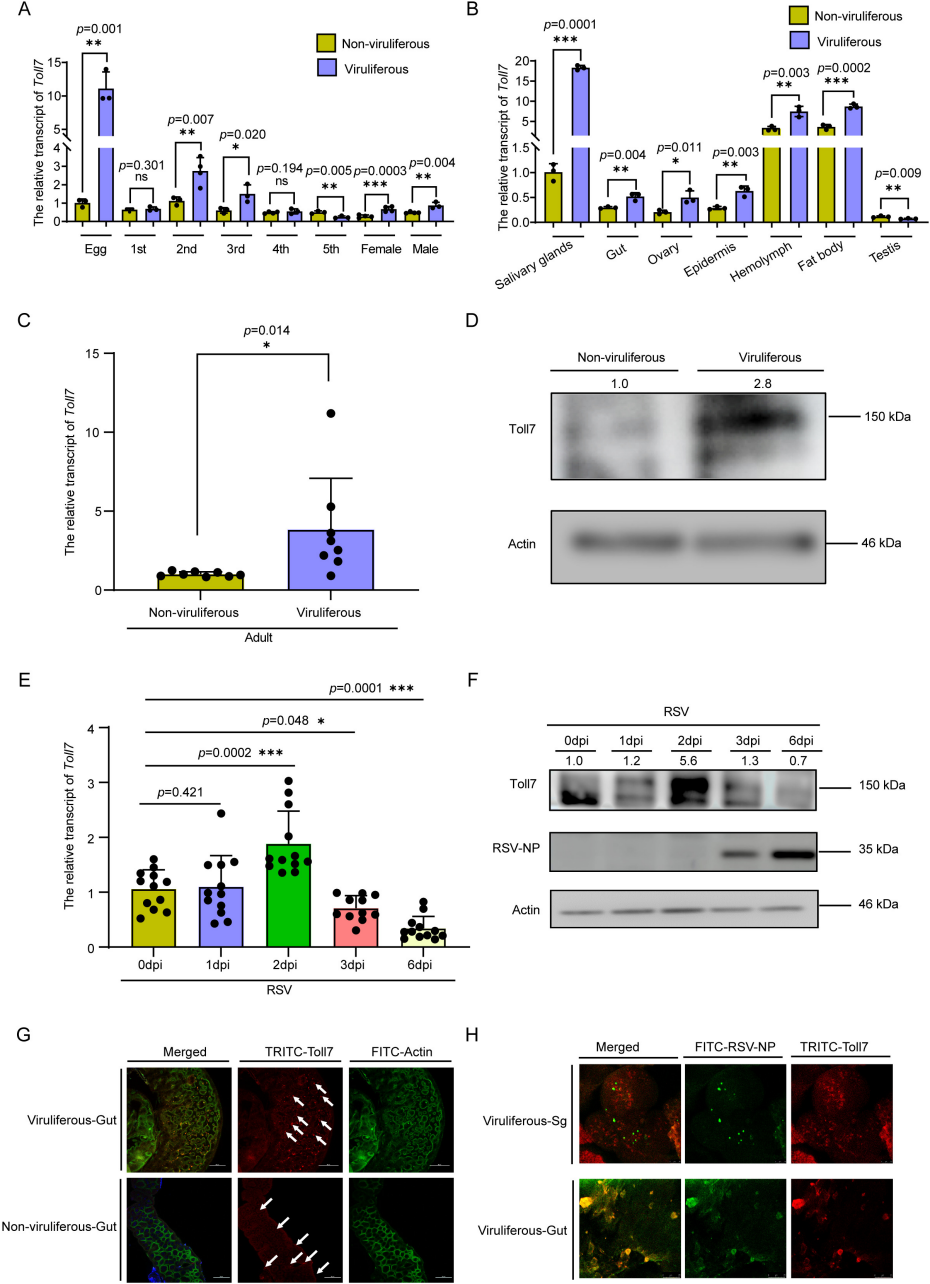
Active response of Toll7 pathway during rice stripe virus (RSV) infection. (A, B) Quantitative analysis of *Toll7* transcript levels across various developmental stages of *Laodelphax striatellus* (eggs, 1st to 5th instar nymphs, and adult females and males) (A) and in distinct tissues (salivary gland, gut, ovary, epidermis, haemolymph, fat body and testis) (B) in both non‐viruliferous and viruliferous planthoppers. Three independent biological replicates were conducted. (C, D) Comparative evaluation of *Toll7* mRNA level (C) and protein level (D) between non‐viruliferous and viruliferous adult planthopper populations. Each data point corresponds to a biological replicate, with eight adult *L. striatellus* individuals sampled per group. (E,F) Temporal transcriptional (E) and protein (F) levels of *Toll7* in *L. striatellus* following RSV infection at specified intervals (0, 1, 2, 3 and 6 days post‐inoculation [dpi]). (G) Immunofluorescent visualization of Toll7 localization within the gut tissue of non‐viruliferous and viruliferous *L. striatellus* . (H) The co‐localization of Toll7 and RSV‐NP in viruliferous *L. striatellus* . Scale bar: 50 μm. Statistical significance was assessed using the *t* test, with asterisks indicating significant differences (*p < 0.05, **p < 0.01, ***p < 0.001). Error bars signify the standard error (SE) of the mean.

The expression profile of *Toll7* was compared between viruliferous and non‐viruliferous *L. striatellus* across different developmental stages and tissues using reverse transcription‐quantitative PCR (RT‐qPCR). The results showed that *Toll7* expression was significantly higher in viruliferous planthoppers than in non‐viruliferous individuals at most developmental stages (*p* < 0.01) and in most tissues (*p* < 0.01) (Figure 1A,B). Specifically, the highest abundance of *Toll7* mRNA was observed in the eggs of viruliferous planthoppers, followed closely by the second instar nymphs (Figure 1A). Moreover, *Toll7* expression was predominantly localized to the salivary glands, where it exhibited the highest levels compared to other tissues in viruliferous *L. striatellus* (Figure 1B). These results indicated the potential role of Toll7 in the physiological and developmental responses of *L. striatellus* to viral infection.

To delineate the potential roles of Toll7 in RSV infection, a comparative analysis of *Toll7* expression profiles was conducted between viruliferous and non‐viruliferous *L. striatellus* . A statistically significant upregulation of *Toll7* expression was observed in both viruliferous adult and third‐instar nymph populations (Figure 1C and Figure S1A). In addition, Toll7 protein levels were significantly higher in viruliferous insects than in non‐viruliferous ones (Figure 1D). Furthermore, temporal expression profiling revealed a pronounced upregulation of *Toll7* at 2 days post‐infection (dpi), which was subsequently followed by a significant attenuation at 3 dpi and 6 dpi relative to the control (0 dpi). No significant alteration in *Toll7* expression was detected at 1 dpi following RSV infection (Figure 1E). The protein levels of Toll7 at different time points also showed similar expression profiles (Figure 1F). Complementary immunolabelling experiments further substantiated these observations, revealing an elevation in Toll7 protein expression and an intensified fluorescent signal (Figure 1G). Subsequently, the salivary glands and midguts of viruliferous insects were probed with antibodies targeting RSV‐NP and Toll7. Co‐localization of Toll7 and RSV was observed. Notably, the fluorescence signal intensity of Toll7 correlated positively with viral accumulation, being stronger in regions with higher viral load (Figure 1H). These findings collectively indicate that Toll7 is subject to dynamic regulation and is potentially activated in response to RSV infection.

### 2.2 Toll7 Plays a Critical Role in Host Defence Against RSV Infection

**FIGURE 2:**
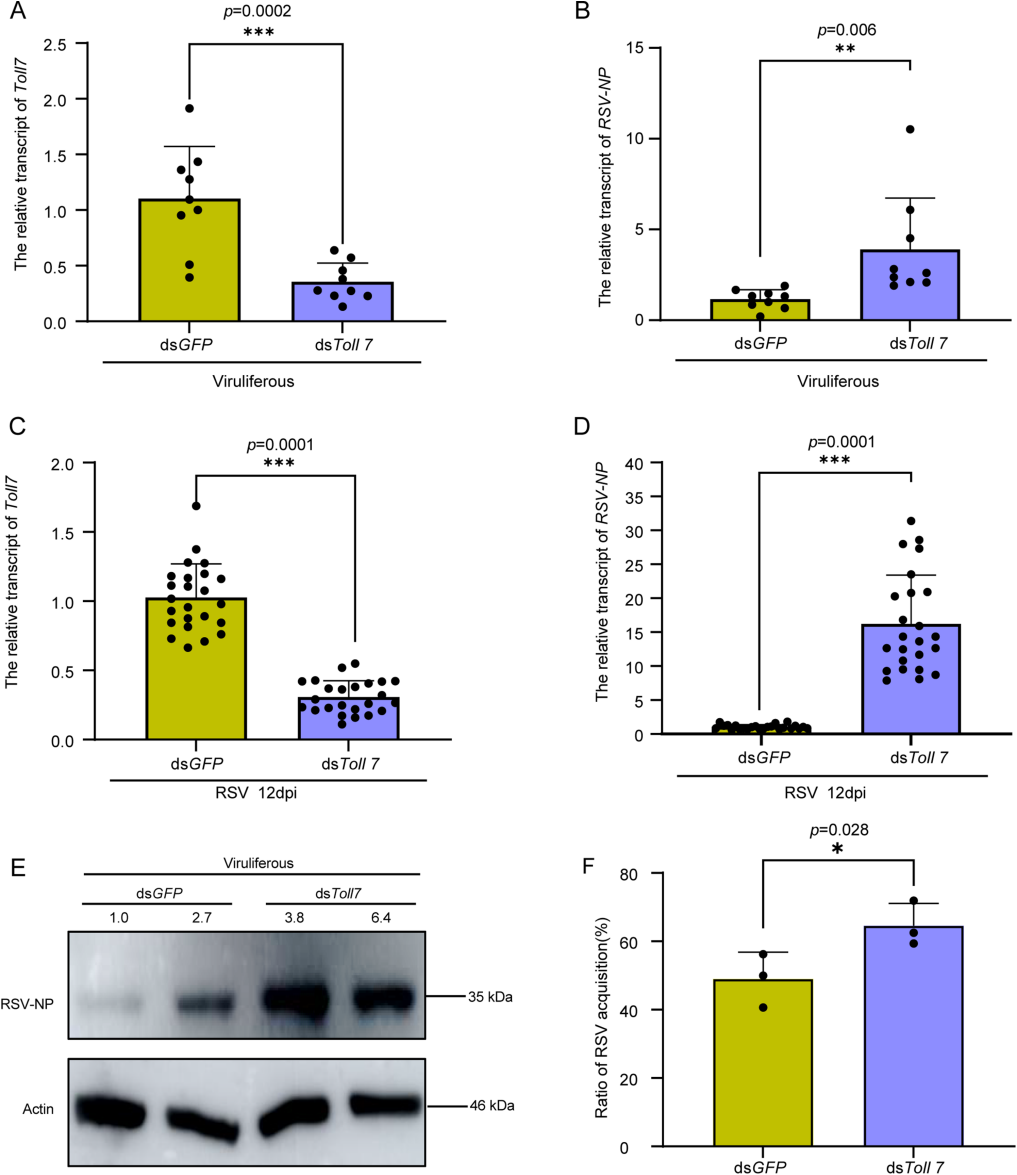
Toll7 plays a critical role in host defence against rice stripe virus (RSV) infection. (A, B) Relative expression profiles of *Toll7* and *RSV‐NP* transcripts in ds*Toll7*‐treated viruliferous *Laodelphax striatellus* . Each data point corresponds to a biological replicate, with 8–10 individuals analysed per group. (C, D) Relative transcript abundance of *Toll7* and *RSV‐NP* in non‐viruliferous *L. striatellus* . Following a 2‐day ds*Toll7* treatment, planthoppers were fed on RSV‐infected rice seedlings for 3 days and subsequently transferred to healthy seedlings for an additional 12‐day feeding period. Each data point represents a biological replicate, with 25–28 individuals sampled per group. (E) Influence of *Toll7* knockdown on RSV‐NP protein accumulation in viruliferous *L. striatellus* after 2 days of ds*Toll7* treatment. Actin antibody was employed to ensure consistent sample loading across treatment groups. Two biological replicates were conducted, each involving 10–15 individuals. (F) Impact of *Toll7* silencing on RSV acquisition efficiency in non‐viruliferous *L. striatellus* at 12 days post‐inoculation. ds*GFP* was used as the control in all experimental setups. Statistical significance was assessed using the *t* test, with *, ** and *** indicating significant differences (*p* < 0.05, *p* < 0.01 and *p* < 0.001, respectively). Error bars depict the standard error (SE) of the mean.

To elucidate the functional significance of Toll7 in RSV‐infected *L. striatellus* , double‐stranded RNA (dsRNA) targeting *Toll7* was synthesized and microinjected into viruliferous planthoppers. The RNA interference (RNAi) efficiency of *Toll7* reached approximately 70% at 2 dpi (Figure 2A). Concurrently, both the relative transcript and protein levels of RSV‐NP were significantly elevated compared to the control group (Figure 2B,E). In a parallel experimental setup, non‐viruliferous *L. striatellus* were subjected to ds*Toll7* injection and, following a 2‐day incubation, allowed to feed on RSV‐infected rice seedlings for 3 days. Subsequently, the insects were transferred to healthy rice seedlings for an additional 12‐day feeding period. RT‐qPCR analysis demonstrated a significant reduction of *Toll7* transcripts, concomitant with a marked upregulation in *RSV‐NP* expression (Figure 2C,D). Additionally, the RSV acquisition rate was quantified using RT‐qPCR, demonstrating a significant increase in the ds*Toll7*‐treated group compared to the ds*GFP* control (Figure 2F). These findings collectively indicate that Toll7 plays a critical role in mediating antiviral responses against RSV infection in *L. striatellus* .

### 2.3 Interaction Between Toll7 of *L. striatellus* and Glycoprotein of RSV

**FIGURE 3:**
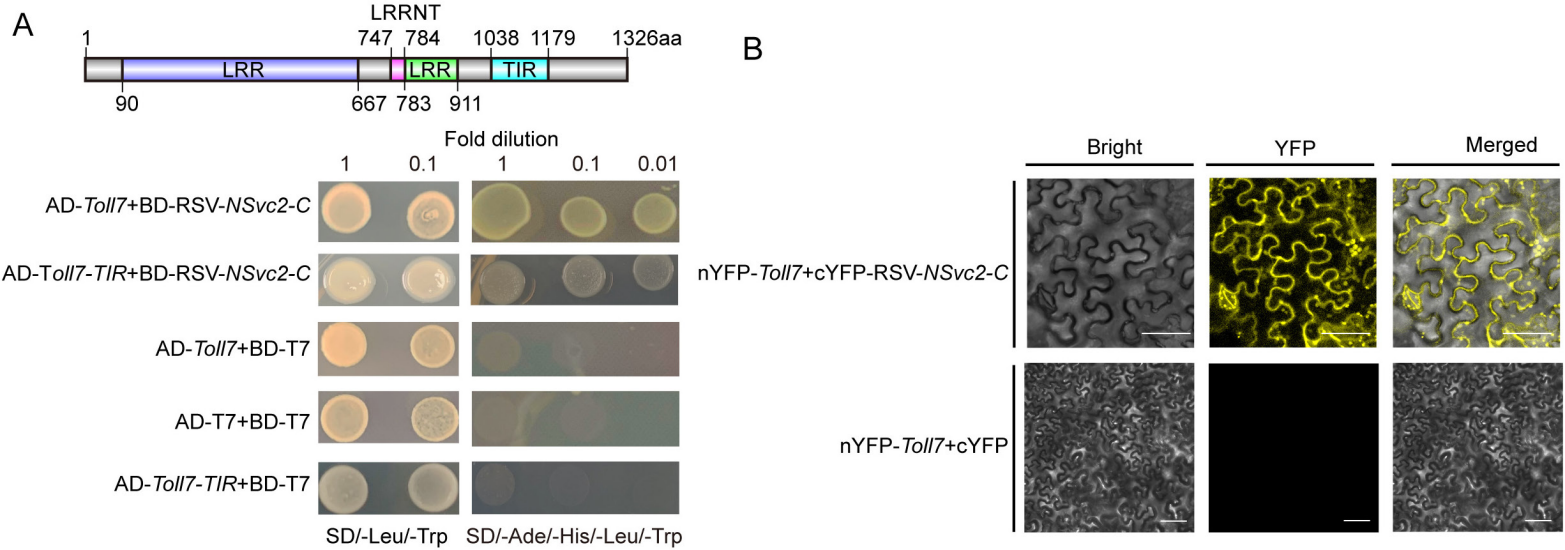
Interaction between Toll7 of *Laodelphax striatellus* and glycoprotein of rice stripe virus (RSV). (A) The interaction between RSV NSvc2‐C and Toll7, as well as the TIR domain (1038–1179 amino acids) of Toll7, was validated using a yeast two‐hybrid assay cultured in SD/−Ade/−His/−Leu/−Trp medium. The images were taken after 3 days incubation at 30°C. (B) Bimolecular fluorescence complementation assay confirmed the interaction between Toll7 and RSV NSvc2‐C in the cytoplasm of *Nicotiana benthamiana* leaves. Pronounced YFP fluorescence signals were detected in the experimental group, indicative of a robust interaction. Scale bars represent 50 μm.

To delineate potential molecular interactions between RSV and Toll7, a yeast two‐hybrid (Y2H) screening assay was employed, using Toll7 and its functional domains (LRR, LRR N‐terminal [LRRNT] and Toll/interleukin‐1 receptor [TIR]) as bait to screen for interactions with RSV‐encoded proteins. The results demonstrated that, among the five RSV proteins evaluated (NSvc2‐C, NSvc2‐N, NS3, NS4 and MP), only NSvc2‐C exhibited a specific interaction with the TIR domain (amino acids 1038–1179) of Toll7, as confirmed by growth on stringent SD/−Leu/−Trp/−His/−Ade selection medium (Figure 3A and Figure S2B). Phylogenetic analysis further demonstrated that Toll7 in *L. striatellus* shares the highest homology with its ortholog in *Nilaparvata lugens* , underscoring the evolutionary conservation of Toll7 (Figure S2A). Bimolecular fluorescence complementation (BiFC) assays were conducted to verify this interaction in *Nicotiana benthamiana* cells. Strong yellow fluorescent protein (YFP) signals were observed in the cytoplasm and cell membrane when nYFP‐*Toll7* and cYFP‐*RSV‐NSvc2‐C* were coexpressed, whereas no fluorescence was detected in the negative control (nYFP‐*Toll7* and cYFP) (Figure 3B). These findings collectively indicate a direct physical interaction between Toll7 and RSV‐NSvc2‐C, suggesting that Toll7 may function as a PRR in *L. striatellus* , recognizing PAMPs during RSV infection.

### 2.4 Downstream Immune Effectors Regulated by the Toll7 Pathway Mediate Anti‐RSV Response in *L. striatellus*

**FIGURE 4:**
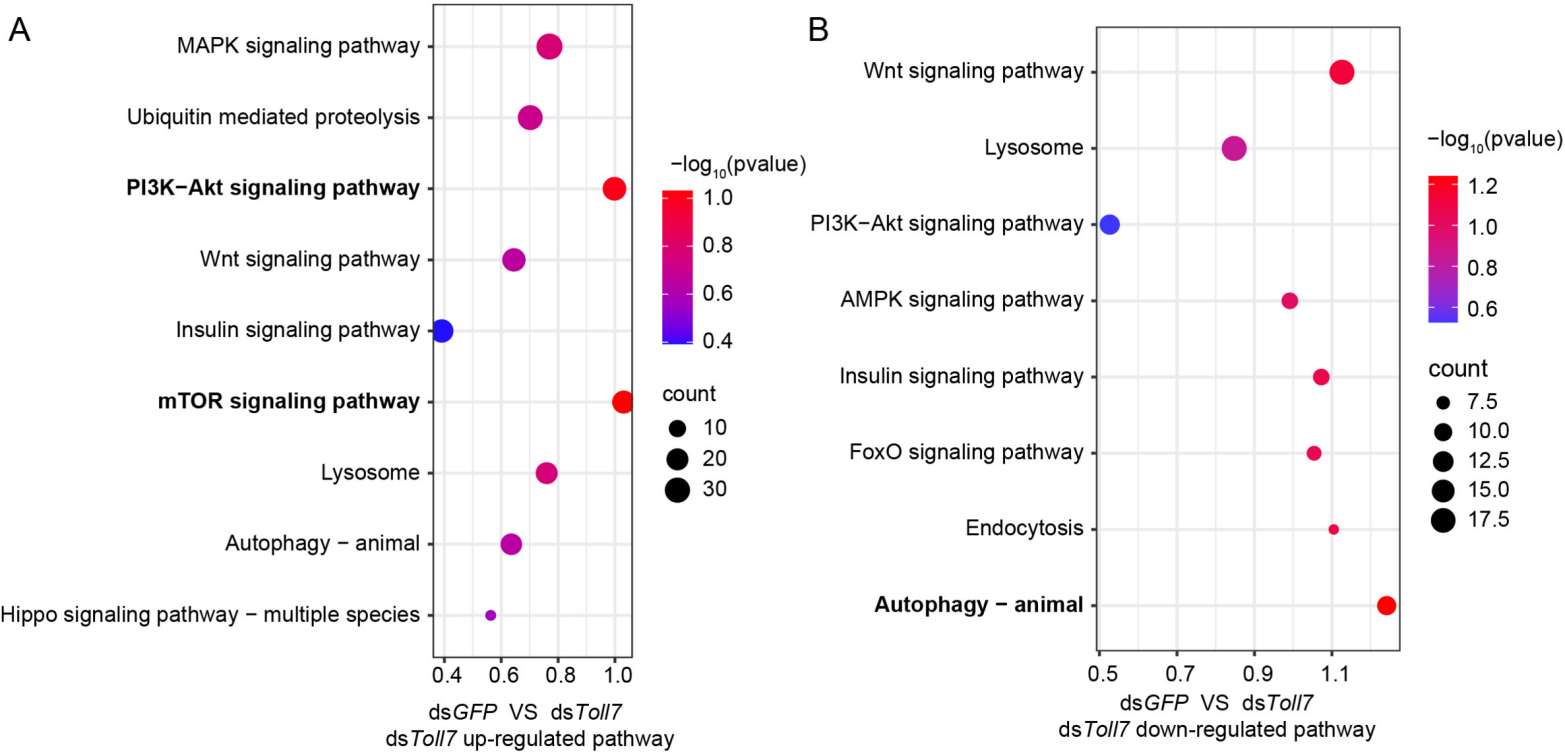
Downstream immune effectors regulated by the Toll7 pathway mediate anti‐rice stripe virus (RSV) response in *Laodelphax striatellus* . (A, B) KEGG pathway enrichment analysis revealing upregulated (A) and downregulated (B) pathways among differentially expressed genes in viruliferous *L. striatellus* following ds*Toll7* treatment. ds*GFP* served as the control. Each experimental group comprised three biological replicates. Pathways were considered significantly altered when the log_2_ fold change ratio was ≥ 1 and *p* ≤ 0.05.

To further delineate the molecular mechanisms underlying Toll7‐mediated antiviral defence, a transcriptomic analysis was performed to identify differentially expressed genes (DEGs) in viruliferous planthoppers following the knockdown of *Toll7*, with ds*GFP*‐treated planthoppers serving as the control group. The KEGG pathway enrichment analysis of the DEGs revealed significant upregulation of two immune‐related pathways: PI3K‐Akt and mTOR (Figure 4A). In contrast, the autophagy pathway, a critical component of cellular immune responses, was markedly downregulated upon *Toll7* silencing (Figure 4B). These results collectively suggest that the PI3K‐Akt, mTOR and autophagy pathways are integral to the Toll7‐regulated antiviral defence mechanisms against RSV infection.

### 2.5 Toll7‐Mediated Negative Regulation of the PI3K‐Akt–mTOR Pathway Modulates Antiviral Responses

**FIGURE 5:**
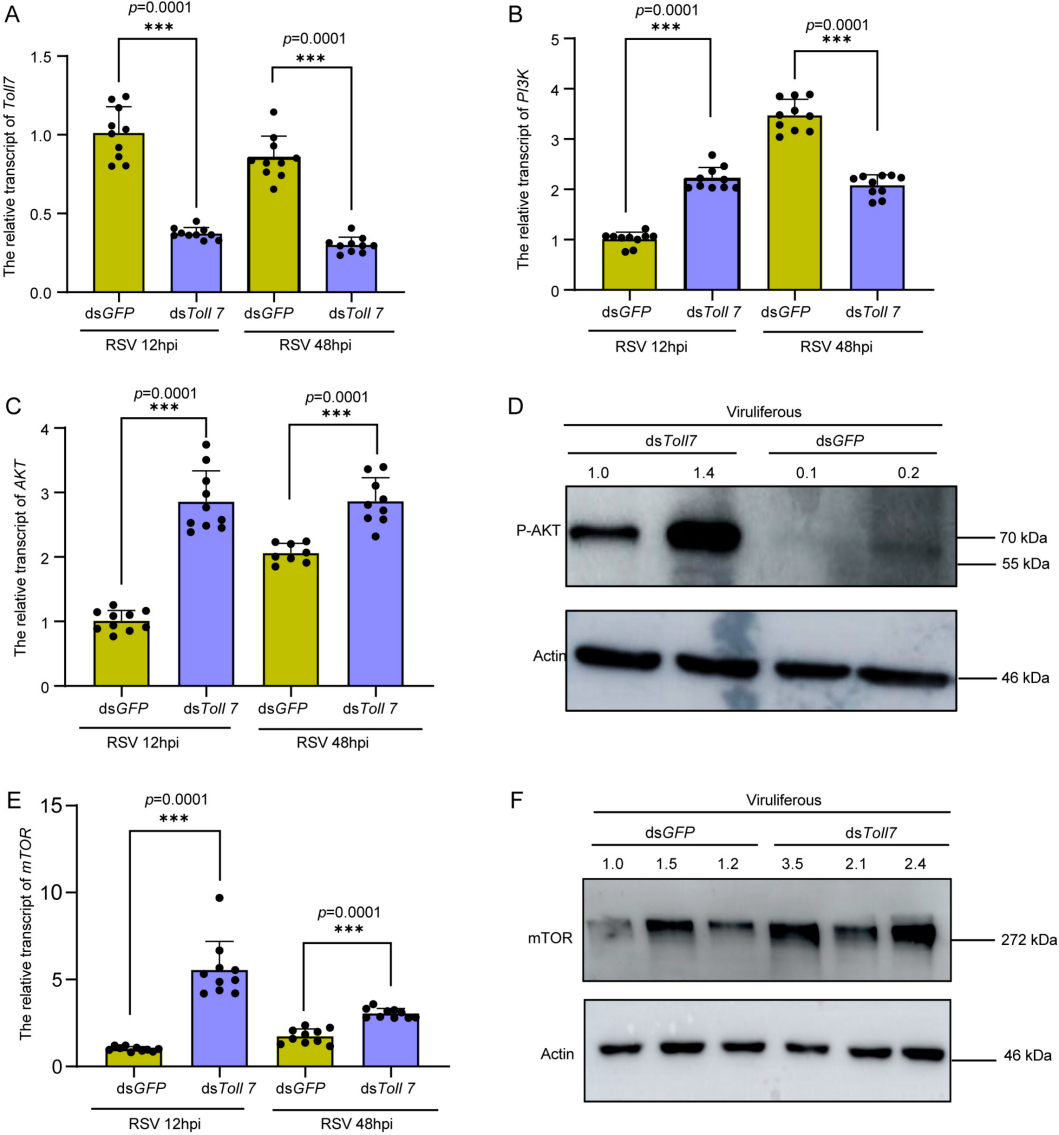
Toll7‐mediated negative regulation of the PI3K‐Akt–mTOR pathway modulates antiviral responses. (A–C, E) Relative transcript levels of *Toll7* (A), *PI3K* (B), *AKT* (C) and *mTOR* (E) in non‐viruliferous *Laodelphax striatellus* following *Toll7* knockdown and subsequent rice stripe virus (RSV) infection at 12 and 48 h post‐inoculation (hpi). Each data point represents a biological replicate, with 8–10 individuals sampled per group. (D, F) Impact of *Toll7* knockdown on the protein levels of phosphorylated Akt (P‐Akt) and mTOR in viruliferous *L. striatellus* treated with ds*Toll7* for 2 days. Actin antibody was used to normalize sample concentrations across treatment groups. Two biological replicates were conducted, each comprising 10–15 individuals. Statistical significance was determined using the *t* test, with *** indicating a significant difference (*p* < 0.001). Error bars depict the standard error (SE) of the mean.

To further validate the regulatory relationship between Toll7 and the PI3K‐Akt–mTOR signalling pathway, the relative transcript levels of *PI3K*, *Akt* and *mTOR* were quantified in non‐viruliferous *L. striatellus* subjected to ds*Toll7* treatment and subsequent RSV infection at 12 and 48 h post‐infection (hpi). The results demonstrated that effective silencing of *Toll7* led to a significant upregulation in the transcription levels of all three genes at 12 hpi, as well as *Akt* and *mTOR* at 48 hpi (Figure 5A–C,E). Conversely, the transcriptional level of *PI3K* exhibited a pronounced downregulation at 48 hpi (Figure 5B). Complementary to these findings, western blot analysis demonstrated a substantial elevation in the protein levels of phosphorylated Akt (P‐Akt) and mTOR in viruliferous *L. striatellus* following *Toll7* knockdown (Figure 5D,F). Collectively, these findings indicate that Toll7 negatively regulates the expression of the PI3K‐Akt–mTOR signalling pathway, thereby modulating the antiviral response in *L. striatellus* .

### 2.6 The PI3K‐Akt–mTOR Pathway Facilitates RSV Replication in *L. striatellus*

**FIGURE 6:**
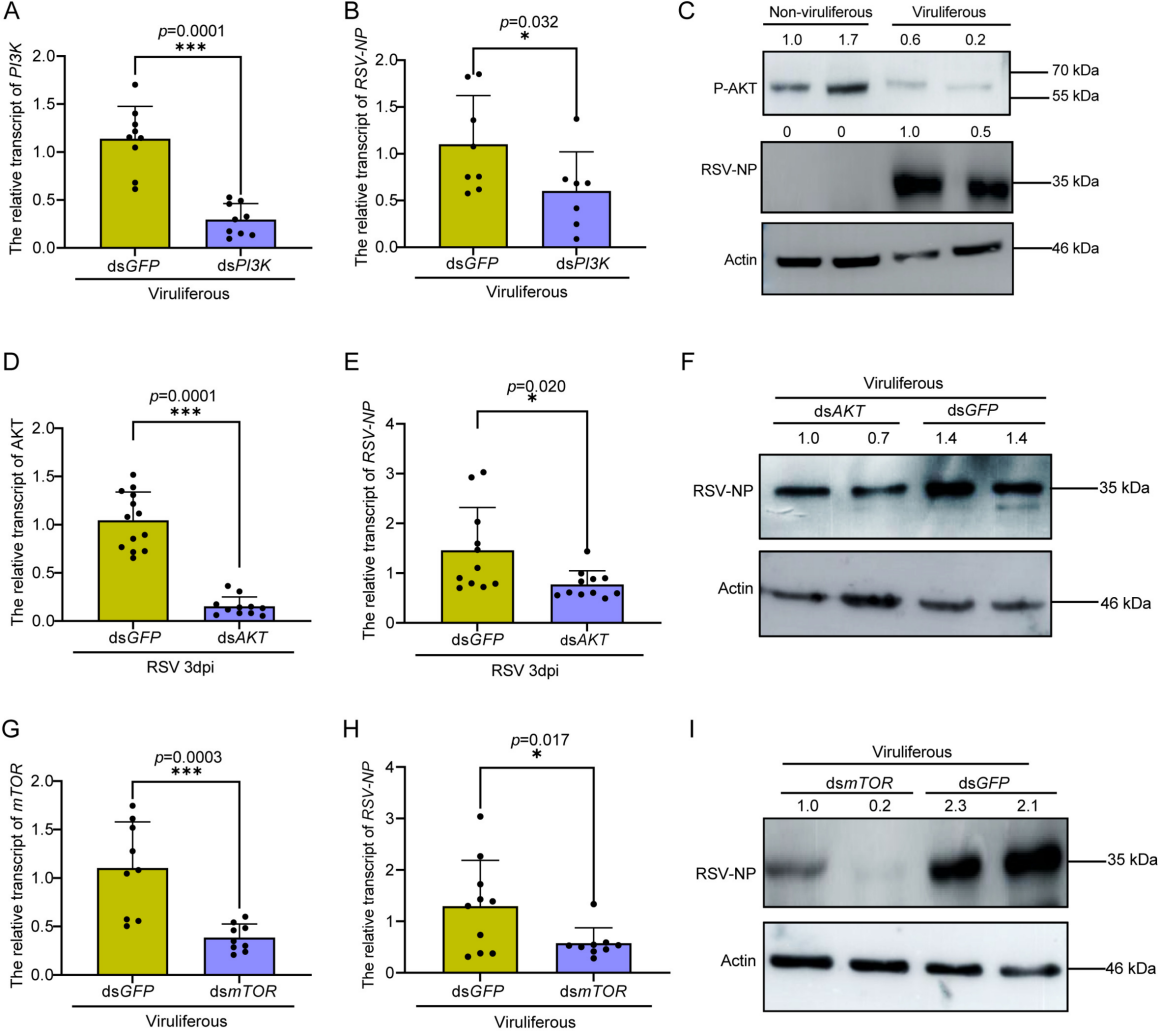
The PI3K‐Akt–mTOR pathway facilitates rice stripe virus (RSV) replication in *Laodelphax striatellus* . (A, B) Relative transcript levels of *PI3K* (A) and *RSV‐NP* (B) in viruliferous *L. striatellus* following *PI3K* knockdown. (C) Comparative analysis of P‐Akt protein levels between non‐viruliferous and viruliferous *L. striatellus* . (D) Silencing efficiency of *AKT* in viruliferous *L. striatellus* treated with ds*Akt*. (E, F) Impact of *Akt* knockdown on the transcription (E) and protein levels (F) of RSV‐NP in viruliferous *L. striatellus* treated with ds*Akt* for 2 days. (G, H) Relative transcript levels of *mTOR* (G) and *RSV‐NP* (H) in viruliferous *L. striatellus* following *mTOR* knockdown. (I) Effect of *mTOR* knockdown on RSV‐NP protein levels in viruliferous *L. striatellus* . For transcript level analysis, each data point represents a biological replicate, with 8–12 individuals sampled per group. For protein level analysis, actin antibody was used to normalize sample concentrations across treatment groups. Two biological replicates were performed, each comprising 10–15 individuals. Statistical significance was assessed using the *t* test, with * indicating a significant difference (*p* < 0.05) and *** denoting an extremely significant difference (*p* < 0.001). Error bars represent the standard error (SE) of the mean.

Given the direct regulatory role of Toll7 in the PI3K‐Akt–mTOR signalling pathway, the potential functional involvement of this pathway in RSV replication was systematically investigated. Transcriptional and protein levels of RSV‐NP were quantified in viruliferous *L. striatellus* following silencing of *PI3K*, *Akt* and *mTOR* for 2 days. The results revealed a significant downregulation in *RSV‐NP* transcript levels upon silencing of *PI3K*, *Akt* and *mTOR* (Figure 6A,B,D,E,G,H), accompanied by a pronounced reduction in RSV‐NP protein levels following *Akt* and *mTOR* knockdown (Figure 6F,I). Additionally, the protein level of P‐Akt was significantly higher in non‐viruliferous compared to viruliferous *L. striatellus* (Figure 6C). To further assess potential direct interactions between mTOR and RSV proteins, a Y2H assay was performed. The results revealed no interaction between the conserved TEL domain of mTOR and RSV proteins (Figure S3A,B). Collectively, these findings provide robust evidence that the PI3K‐Akt–mTOR pathway facilitates RSV replication in *L. striatellus* .

### 2.7 Toll7 Positively Regulates the Autophagy Pathway in *L. striatellus*

**FIGURE 7:**
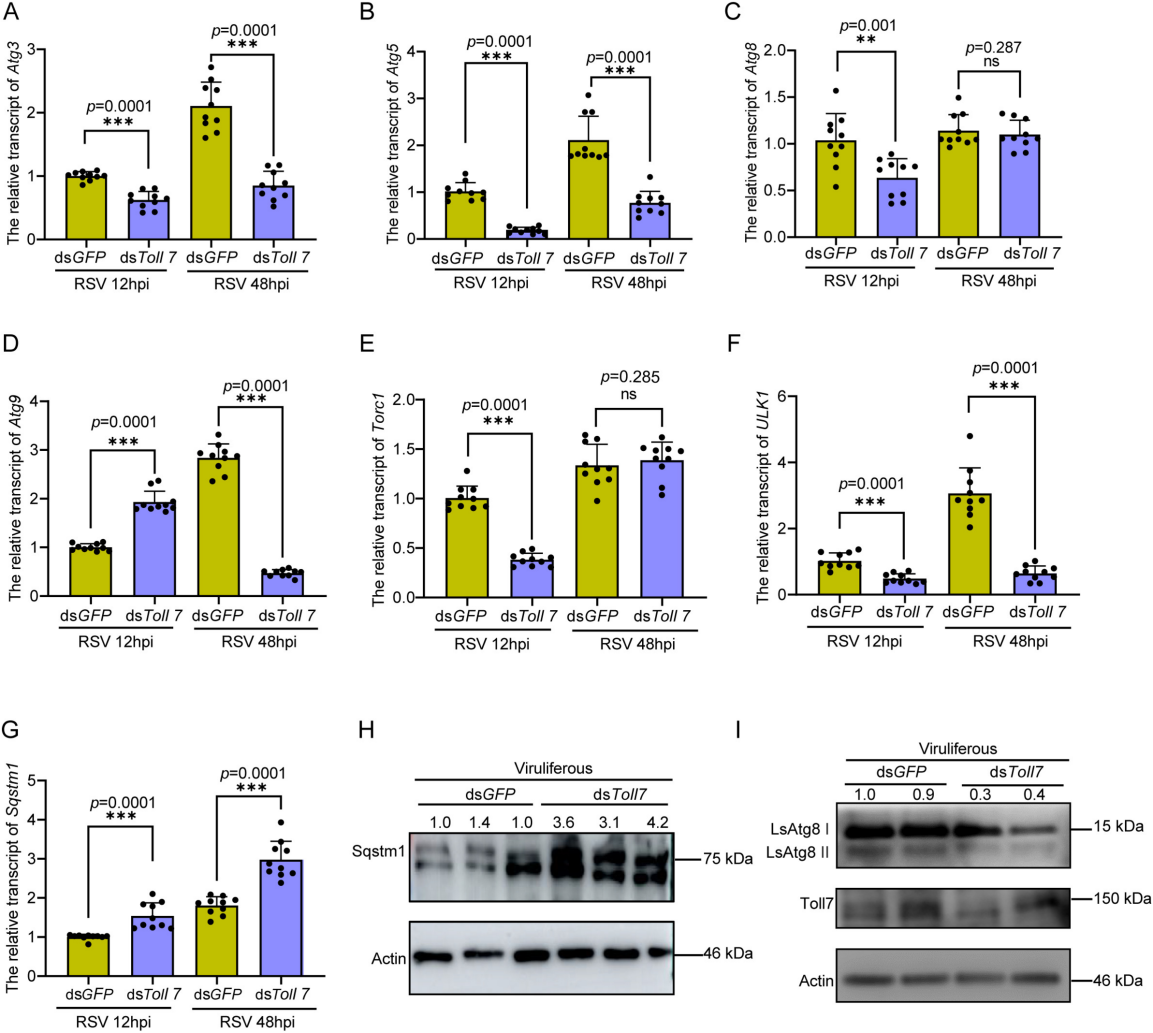
Toll7 positively regulates the autophagy pathway in *Laodelphax striatellus* . (A–G) Relative transcript levels of autophagy‐related genes, including *Atg3* (A), *Atg5* (B), *Atg8* (C), *Atg9* (D), *Torc1* (E), *ULK1* (F) and *Sqstm1* (G), in non‐viruliferous *L. striatellus* following *Toll7* knockdown and subsequent rice stripe virus (RSV) infection at 12 and 48 h post‐inoculation (hpi). Each data point represents a biological replicate, with 8–10 individuals sampled per group. (H) Impact of *Toll7* knockdown on the protein levels of Sqstm1 in viruliferous *L. striatellus* . (I) Effect of *Toll7* silencing on ATG8‐I and ATG8‐II protein abundance in viruliferous *L. striatellus* . Actin antibody was used to normalize sample concentrations across treatment groups. Two biological replicates were conducted, each comprising 10–15 individuals. Statistical significance was determined using the *t* test, with *** indicating a significant difference (*p* < 0.001) and ns denoting no significance. Error bars depict the standard error (SE) of the mean.

Previous studies have demonstrated that autophagy plays an active role in the host antiviral response against various viruses, including RSV (He et al. 2024). Based on transcriptome sequencing results, we hypothesized that Toll7 contributes to anti‐RSV defence by modulating the autophagy pathway. To elucidate this mechanism, the relative transcript levels of key autophagy‐related genes, including *Atg3*, *Atg5*, *Atg8*, *Atg9*, *Torc1*, *ULK1* and *Sqstm1*, were measured in non‐viruliferous *L. striatellus* treated with ds*Toll7* and infected with RSV at 12 and 48 hpi. The results revealed that the transcription levels of *Atg3*, *Atg5*, *Atg8*, *Torc1* and *ULK1* exhibited a marked decline at 12 hpi (Figure 7A–C,E,F). In contrast, the expression of *Atg9* displayed a transient upregulation at 12 hpi, followed by a notable reduction at 48 hpi (Figure 7D). Additionally, both the transcriptional and protein levels of *Sqstm1*, a pivotal negative regulator of autophagy, were significantly upregulated (Figure 7G,H). More importantly, ATG8 serves as a key marker protein for autophagy. During the induction of autophagy, the conversion from ATG8‐I to ATG8‐II is a required indicator. Therefore, the protein level of ATG8 was also measured, and a significant decrease in ATG8‐II was observed upon *Toll7* silencing (Figure 7I and Figure S4). In conclusion, these findings suggest that Toll7 is involved in anti‐RSV defence by positively regulating the autophagy pathway in *L. striatellus* .

## 3 Discussion

**FIGURE 8:**
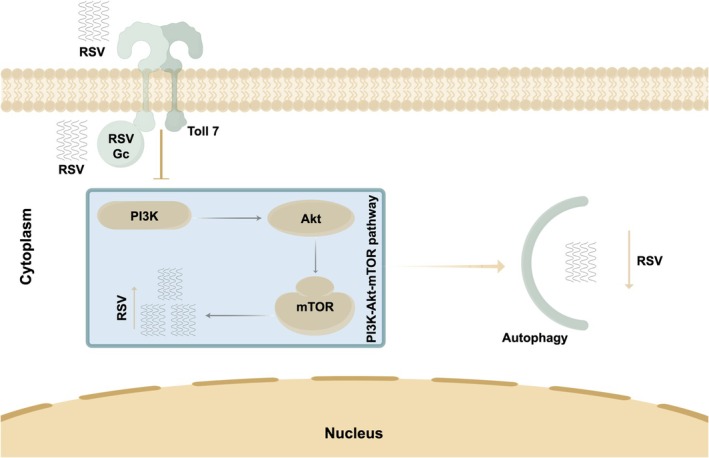
Schematic diagram illustrating the induction of autophagy in *Laodelphax striatellus* by rice stripe virus (RSV) glycoprotein through activation of the Toll7 immune pathway. This study elucidates a novel molecular mechanism wherein RSV glycoprotein Gc interacts with the TIR domain of Toll7, leading to the activation of the Toll7 receptor. This interaction subsequently inhibits the intracellular PI3K‐Akt‐TOR signalling cascade, thereby inducing autophagy. This autophagic response plays a critical role in inhibiting persistent RSV infection in *L. striatellus* . The schematic was created using Figdraw software.

Through long‐term evolution, insect vectors have developed multiple antiviral strategies to resist virus infection. Arboviruses have also evolved various counterstrategies to ensure their persistent transmission within insect vectors (Jia et al. 2024; He et al. 2024). Generally, the canonical innate Toll‐Dorsal immune signalling pathway plays a crucial role in the antiviral defence mechanisms in invertebrates, such as *Drosophila* (Ferreira et al. 2014). Our previous studies have also demonstrated the existence of a Toll‐Dorsal‐ZN708 signalling cascade in insect vectors, which mediates antiviral immune responses (He et al. 2024). However, the arboviruses activate different Toll receptors during the infection of insect vectors, and the resulting diverse signalling cascades still require more comprehensive investigation. In the present study, as illustrated in the schematic diagram (Figure 8), we elucidated a new molecular mechanism by which an arbovirus activates the Toll7 protein in cells of insect vectors, which inhibits the intracellular PI3K‐Akt‐TOR signalling cascade, and induces autophagy, thereby protecting against persistent infection of RSV in *L. striatellus* .

The recognition of pathogens by Toll receptors on the cell membrane can occur in different forms: Toll receptors can directly function as PRRs to recognize PAMPs (Medzhitov 2001; Werling and Jungi 2003; Delgado and Deretic 2009), or they can be activated indirectly through pathogen‐induced responses, such as proteolytic cascades that cleave Spätzle (Hu et al. 2004; Weber et al. 2003). As a crucial member of the Toll receptors family, Toll7 receptor acts as a PRR, primarily recognizing viral PAMPs (Delgado and Deretic 2009; Nakamoto et al. 2012; Jia et al. 2024; Moy et al. 2014; Lund et al. 2004). In insect vectors, Toll7 has been reported to function as a PRR recognizing insect‐borne viruses during SRBSDV infection in *Sogatella furcifera* , although the specific PAMPs of SRBSDV have not been clearly identified (Jia et al. 2024). Surprisingly, our study revealed that Toll7 does not function as a canonical receptor in recognizing RSV. Rather, RSV intracellularly binds to the TIR domain of Toll7 via its glycoprotein, triggering downstream signalling cascades that suppress persistent viral infection (Figures 1, 2, 3). Although Toll7/TLR7 acts as a receptor for viral recognition during VSV infection in *Drosophila* and RVFV infection in mammalian cells—further substantiating the critical role of viral glycoproteins in engaging Toll/TLR7—their binding modalities with viral glycoproteins may diverge significantly due to evolutionary divergence between these receptors (Nakamoto et al. 2012; Cherry 2009; Moy et al. 2014; Leulier and Lemaitre 2008). In summary, contrary to the canonical paradigm where Toll‐like receptors recognize viral glycoproteins via extracellular domains, we demonstrate a non‐canonical activation pathway: the glycoprotein of an insect‐vectored plant virus directly engages the TIR domain of Toll7 to trigger downstream signalling cascades.

Activated Toll7 initiates multiple downstream signalling pathways, such as the canonical Toll‐Dorsal signalling pathway (Jia et al. 2024) and the PI3K‐Akt–mTOR signalling pathway (Cherry 2009; Moy et al. 2014). This study demonstrates that in RSV‐infected *L. triatellus* , knocking down *Toll7* significantly upregulated the expression of genes associated with the PI3K‐Akt–mTOR signalling pathway (Figure 5). Silencing *PI3K*, *Akt* and *mTOR* promoted RSV infection in *L. striatellus* (Figure 6). In *Drosophila*, Toll7 recognition of VSV leads to the suppression of the PI3K‐Akt–TOR signalling pathway (Cherry 2009). Similarly, in mammalian cells, TLR7 recognition of RVFV results in a significant reduction in intracellular Akt levels (Moy et al. 2014). We have elucidated that Toll7 in insect vectors, upon interacting with the insect‐borne virus RSV, suppresses the downstream PI3K‐Akt–mTOR signalling cascade within cells. While the binding modes of viruses to Toll/TLR7 differ, the resulting changes in downstream signalling cascades are conserved across species.

As an evolutionarily conserved protein kinase, mTOR regulates cell growth and autophagy by sensing nutrient status, stress and growth factor signals (Jung et al. 2010). Typically, TOR exerts a negative regulatory effect on autophagy. That is, under conditions of nutrient deprivation, reduced energy availability or stress, the activity of TOR decreases, alleviating its inhibition on autophagy‐related proteins and thereby promoting the initiation of autophagy (Jung et al. 2010; Wang and Zhang 2019). By knocking down *Toll7* in RSV‐infected *L. striatellus* , a significant upregulation of genes related to the mTOR signalling cascade, while the expression of autophagy‐promoting genes (*Atg3*, *Atg5*, *Atg8*, *Torc1*, *ULK1*) was significantly downregulated, and the expression of autophagy‐inhibiting genes (*Sqstm1*) was significantly upregulated (Figures 6 and 7). These experimental results precisely demonstrate that mTOR exerts a negative regulatory effect on autophagy in insect vectors. Furthermore, autophagy plays a critical role in antiviral mechanisms. Our previous studies have also shown that autophagy, as an important immune effector pathway, restricts viral replication and spread, providing essential protection against viral infections (He et al. 2024).

In summary, upon recognition of RSV by the TIR domain of Toll7 in *L. striatellus* , the PI3K‐Akt–mTOR signalling cascade is suppressed. This subsequently leads to the significant activation of autophagy‐promoting genes and the inhibition of autophagy‐suppressing genes. As a result of autophagy activation, the replication and proliferation of RSV within *L. striatellus* are inhibited, ultimately enabling the insect to suppress persistent viral infection. Our findings provide new insights into how vector insects use intracellular signalling cascades to resist persistent viral infections. However, future studies are needed to further elucidate the specific mechanisms by which mTOR regulates autophagy and how autophagy suppresses persistent viral infections in insect vectors.

## 4 Experimental Procedures

### 4.1 Insects and Viruses

The experimental cohorts of *L. striatellus* , encompassing both non‐viruliferous and viruliferous individuals, were cultivated on susceptible rice seedlings (cultivar Wuyujing No. 3) within a controlled environmental chamber. The chamber was regulated to maintain a constant temperature of 25°C ± 1°C, a relative humidity range of 70%–80% and a photoperiodic cycle of 14 h of light followed by 10 h of darkness. The infection prevalence of RSV within the planthopper population was ascertained to be approximately 80%. This infection rate was routinely verified every three to four generations through the application of RT‐PCR assays, as delineated in previous studies (Li et al. 2013; He et al. 2021).

### 4.2 Total RNA Extraction and qPCR Analysis

Total RNA was isolated from insect samples using TRIzol reagent (TaKaRa) following the manufacturer's protocol. The integrity and concentration of the extracted RNA were subsequently evaluated using a NanoDrop spectrophotometer (Thermo Scientific) to ensure high‐quality RNA for downstream applications. For cDNA synthesis, 1 μg of total RNA was reverse‐transcribed using the HiScript II Q RT SuperMix for qPCR (+gDNA wiper) (Vazyme), adhering to the prescribed protocol to generate first‐strand cDNA.

Quantitative real‐time PCR (qPCR) was performed in a 10 μL reaction volume, which included 0.5 μL of cDNA template, 5 μL of Hieff qPCR SYBR Green PCR Master Mix (YESEN), 0.2 μL each of 1 μM forward and reverse primers and 4.1 μL of nuclease‐free double‐distilled water. Amplification was carried out on a LightCycler 480 II instrument (Roche) under the following thermal cycling conditions: initial denaturation at 95°C for 5 min; followed by 40 cycles at 95°C for 30 s, 60°C for 30 s and 70°C for 30 s. Relative quantification of gene expression was determined using the 2^−ΔΔ*C*t^ method. Statistical analyses were performed to assess significance, with thresholds set at *p* < 0.05 (*), *p* < 0.01 (**) and *p* < 0.001 (***) as determined by the *t*test.

### 4.3 Spatiotemporal Expression Analysis of the *Toll7* in *L. striatellus*

Samples of *L. striatellus* encompassing eight distinct developmental stages (egg, first to fifth instar nymphs, female and male adults) and seven diverse tissues (salivary gland, gut, ovary, epidermis, haemolymph, fat body and testis) were collected from both non‐viruliferous and viruliferous populations. For the isolation of haemolymph and fat body, planthoppers were dissected in phosphate‐buffered saline (PBS), followed by centrifugation at 5000 *g* for 5 min at 4°C. The supernatant, containing haemolymph and the precipitate, containing fat body, were collected separately. The experimental design incorporated three independent biological replicates. For the collection of samples across different developmental stages, the number of insects varied according to the required sample size for each replicate. For tissue‐specific samples, each replicate comprised tissues pooled from approximately 40–50 individual adult planthoppers to ensure sufficient material for analysis.

### 4.4 Expression Profiles of *Toll7* in *L. striatellus* Following RSV Infection at Various Time Points

To elucidate the expression dynamics of *Toll7* in response to RSV infection, RNA extraction was performed on individual planthoppers from both non‐viruliferous and viruliferous populations. Specifically, approximately eight adult planthoppers or 20–25 third‐instar nymphs were collected for each population. To investigate the temporal expression patterns of *Toll7* during RSV infection, a cohort of approximately 200 second‐instar nymphs from a non‐viruliferous population was allowed to feed on RSV‐infected rice seedlings for a period of 48 h. Subsequently, the planthoppers were transferred to healthy rice seedlings and sampled at designated time points (1, 2, 3 and 6 dpi). At each time point, 12–15 planthoppers were collected and the expression levels of *Toll7* were quantified using RT‐qPCR as previously described.

### 4.5 Evaluation of RSV Acquisition Rates in *L. striatellus*

To determine the RSV acquisition rates in *L. striatellus* , a cohort of 100–200 non‐viruliferous *L. striatellus* individuals was subjected to microinjection with either ds*Toll7* or ds*GFP*. Following a 48‐h incubation period, the insects were provided access to RSV‐infected rice seedlings for a 72‐h acquisition feeding phase. Subsequently, the insects were transferred to healthy rice seedlings and maintained for an additional 12‐day inoculation feeding period. The relative expression levels of *Toll7* and *RSV‐NP* were quantified using RT‐qPCR. The RSV acquisition rate was assessed by detecting viral presence in individual *L. striatellus* through RT‐PCR. The impact of *Toll7* knockdown on the expression profiles of genes associated with the PI3K‐Akt–mTOR signalling pathway and autophagy‐related genes was evaluated at 12 and 48 hpi using RT‐qPCR.

### 4.6 Synthesis of dsRNA and Microinjection Procedure

Gene‐specific primers, incorporating T7 promoter sequences, were used to amplify the *Toll7*, *PI3K*, *Akt* and *mTOR* gene fragments derived from *L. striatellus* . A corresponding fragment of *GFP* gene was employed as a control. The primer sequences used for amplification are detailed in Table S1. dsRNA was synthesized employing the T7 RiboMAX Express RNAi System (Promega) in accordance with the manufacturer's protocol. The integrity and quality of the synthesized dsRNA were assessed via agarose gel electrophoresis. Microinjection of dsRNA was performed by administering 40 nL of dsRNA solution into the ventral thoracic region of individual planthopper using a finely calibrated glass needle, as previously described (He et al. 2021; Xu et al. 2015). Post‐injection, the relative expression levels of *Toll7*, *PI3K*, *Akt*, *mTOR*, *RSV‐NP* and autophagy‐associated genes were quantified using RT‐qPCR.

### 4.7 Gene Cloning and Phylogenetic Analysis

The *Toll7* gene was identified from the transcriptome of *L. striatellus* (Accession Number: SRR4020768) through a homology‐based search, using the corresponding gene sequences from *Nilaparvata lugens* (*NlToll7*, XP_022199456.1) as query sequences. Conserved protein domains of Toll7 and mTOR were predicted using the NCBI Conserved Domains Database (https://www.ncbi.nlm.nih.gov/Structure/cdd/wrpsb.cgi). Phylogenetic analysis was conducted using the deduced amino acid sequences, with phylogenetic trees constructed in MEGA 7.0 software employing the maximum‐likelihood (ML) algorithm. Bootstrap analysis was performed with 1000 replicates to assess the robustness of the tree topology. The full‐length open reading frame (ORF) of *Toll7* was amplified from planthopper cDNA using gene‐specific primer pairs (Table S1) via RT‐PCR. The amplified product was subsequently validated by Sanger sequencing (Sangon Biotech, China) to confirm its accuracy and integrity.

### 4.8 Transcriptome Sequencing and KEGG Enrichment Analysis

Total RNA was isolated from viruliferous *L. striatellus* subjected to ds*Toll7* and ds*GFP* treatments. The integrity and concentration of the extracted RNA were meticulously assessed using a NanoDrop spectrophotometer (Thermo Scientific) to verify the attainment of high‐quality RNA samples. Subsequently, RNA meeting the stringent quality criteria was subjected to transcriptome sequencing, which was conducted by Novogene (China). Raw sequencing data were quality‐checked using FastQC, and low‐quality reads and adapter sequences were removed using Trimmomatic. High‐quality reads were aligned to the reference genome using HISAT2, and transcript assembly and quantification were performed using StringTie. DEGs were identified using DESeq2, with screening criteria set as log_2_fold change > |1| and adjusted *p*‐value (*p*_adj_) < 0.05. To explore the biological functions and metabolic pathways of the DEGs, KEGG (Kyoto Encyclopedia of Genes and Genomes) pathway enrichment analysis was conducted. Significantly enriched pathways were identified with *p* < 0.05, and multiple testing correction was performed using the Benjamini–Hochberg method. The enrichment results were visualized using bubble plots to highlight the significantly enriched immune pathways and their associated genes.

### 4.9 Expression, Purification and Antibody Preparation of Recombinant Proteins

The full‐length ORF of *RSV‐NP* was subcloned into the pET‐28a expression vector to generate a recombinant protein fused with a His‐tag. The specific primer sequences used for cloning are detailed in Table S1. The resultant recombinant plasmid was subsequently transformed into *Escherichia coli* BL21 (DE3) competent cells. Transformed bacterial cultures were incubated at 37°C under constant agitation at 220 rpm until the optical density at 600 nm (OD_600_) reached 0.6–0.8. Protein expression was induced by the addition of 1 mM isopropyl β‐D‐1‐thiogalactopyranoside (IPTG) at a 1:1000 dilution, followed by incubation at 16°C for 8 h. Cells were harvested by centrifugation at 8000 *g* for 10 min at 4°C, and the resulting pellet was resuspended in ice‐cold PBS. Cell lysis was achieved via sonication on ice for 30 min. The His‐tagged recombinant protein was subsequently purified from the clarified supernatant using Ni‐NTA agarose resin (Qiagen) in accordance with the manufacturer's protocol. Polyclonal antibodies against the purified RSV‐NP protein were generated in rabbits by Huabio (Hangzhou, China). Additionally, specific polypeptide sequences derived from the Toll7 (SLDLSRNDLRSLPDC) and Sqstm1 (CSRGGEERKSKGDEG) protein in *L. striatellus* were designed and synthesized for antibody production by GenScript (Nanjing, China). Commercially available antibodies targeting phosphorylated Akt (p‐Akt), mTOR and actin were procured from Huabio. Secondary antibodies, including horseradish peroxidase (HRP)‐conjugated goat anti‐rabbit IgG and goat anti‐mouse IgG, were also obtained from Invitrogen.

### 4.10 Immunohistochemistry

The midguts of both non‐viruliferous and viruliferous *L. striatellus* were meticulously dissected in ice‐cold PBS to maintain tissue integrity. Subsequently, the dissected tissues were fixed in 4% paraformaldehyde at ambient temperature for a duration of 4 h to ensure optimal preservation of cellular structures. Following fixation, the samples were subjected to three sequential washes, each lasting 5 min, using PBS supplemented with 0.1% Tween 20 (PBST) to remove residual fixative. To enhance cellular permeability, the tissues were treated with 2% Triton X‐100 for 30 min, followed by another series of three 5‐min washes with PBST. For immunolabelling, the samples were incubated overnight at 4°C with a polyclonal fluorescent‐conjugated primary antibody specific to Toll7, diluted at a ratio of 1:100 in 1% bovine serum albumin (BSA) to minimize non‐specific binding. Post‐incubation, unbound primary antibodies were removed through three additional 5‐min washes with PBST. Subsequently, the samples were incubated for 1 h at room temperature with secondary antibodies, including TRITC‐ and FITC‐conjugated anti‐actin antibodies, each diluted 1:100 in 1% BSA. Finally, the samples were washed three times with PBST to eliminate unbound secondary antibodies. Fluorescence imaging was performed using an SP8 X confocal microscope (Leica) to capture high‐resolution images of the immunostained tissues.

### 4.11 Western Blotting Analysis

Protein extraction from *L. striatellus* was conducted using lysis buffer, followed by separation via 8%–15% SDS‐PAGE. The resolved proteins were subsequently transferred onto a polyvinylidene fluoride (PVDF) membrane using the eBlot L1 transfer system (Genscript), preceded by a brief immersion in methanol for 10 s. Post‐transfer, the PVDF membrane was subjected to blocking with 5% (w/v) skimmed milk powder prepared in PBST for 2 h at ambient temperature. Primary antibodies, diluted at a ratio of 1:5000 in blocking buffer, were applied to the membrane and incubated for 2 h at room temperature or overnight at 4°C. Following primary antibody incubation, the membrane was probed with HRP‐conjugated secondary antibodies (goat anti‐rabbit IgG HRP or goat anti‐mouse IgG HRP) at a dilution of 1:10,000 in fresh blocking buffer, with incubation carried out for 1 h at room temperature under gentle agitation. The membrane was subsequently washed three times with PBST to remove unbound antibodies. Protein detection was achieved using the SuperSignal West Pico PLUS Chemiluminescent Substrate (Thermo Scientific) in accordance with the manufacturer's protocol, and chemiluminescent signals were captured using the Amersham Imager 680 (Cellular Technology Ltd.).

### 4.12 Y2H Assay

The *Toll7* gene and its conserved domains from *L. striatellus* were cloned into the activation domain of the yeast vector pGAD‐T7. The conserved TEL domain of mTOR was inserted into the DNA‐binding domain of the vector pGBD‐T7 to generate bait plasmids. Additionally, the full‐length sequences of NSvc2‐C, NSvc2‐N, NS3, NP, NS4 and MP were cloned into pGAD‐T7 and pGBD‐T7, respectively. Toll7, its conserved domains, or the TEL domain of mTOR were co‐transformed with RSV‐encoded proteins into Y2Hgold yeast competent cells. The transformed yeast cells were plated on selective synthetic dropout (SD) medium lacking leucine and tryptophan (SD/−Leu/−Trp) to ensure the presence of both plasmids. Protein–protein interactions were subsequently assessed by growing the yeast cells on stringent selective medium lacking adenine, histidine, leucine and tryptophan (SD/−Ade/−His/−Leu/−Trp). Following a 3‐day incubation period at 30°C, images were captured to document the results. Positive interactions were confirmed by the growth of yeast colonies on the SD/−Ade/−His/−Leu/−Trp medium.

### 4.13 BiFC Assay

To further validate the protein–protein interaction, the full‐length coding sequence of the RSV protein NSvc2‐C was amplified via PCR and subsequently cloned into the pCV‐cYFP expression vector. Similarly, the full‐length ORF of *Toll7* was cloned into the pCV‐nYFP expression vector. The constructed pCV‐nYFP‐*Toll7* and pCV‐cYFP‐*NSvc2‐C* were introduced into *Agrobacterium tumefaciens* GV3101 using a heat‐shock transformation protocol. Subsequently, the recombinant *Agrobacterium* strains harbouring pCV‐nYFP‐*Toll7* and pCV‐cYFP‐*NSvc2‐C* were co‐infiltrated into leaves of *N. benthamiana* for transient expression. The reconstitution of YFP fluorescence, indicative of protein interaction, was visualized and captured using a confocal laser scanning microscope (Nikon).

## Author Contributions

**Yu‐Juan He:** conceptualization, methodology, data curation, investigation, funding acquisition, validation, writing – original draft, writing – review and editing. **Chuan‐Xi Zhang:** funding acquisition. **Qian‐Zhuo Mao:** methodology. **Lin Lin:** project administration. **Jian‐Ping Chen:** resources, funding acquisition, project administration, conceptualization, writing – review and editing. **Xiao‐Wei Wang:** methodology. **Yu‐Hua Qi:** investigation. **Gang Lu:** methodology. **Gao‐Yang Jiao:** investigation. **Can Wang:** investigation. **Zhuang‐Xin Ye:** software, formal analysis. **Jun‐Min Li:** conceptualization, methodology, funding acquisition, writing – review and editing, resources.

## Funding

This project was supported by funds from Ningbo Public Welfare Research Fund (2024S106), Ningbo Natural Science Foundation (2024J180), the China Postdoctoral Science Foundation (2023M741836), the Natural Science Foundation of Zhejiang Province (ZCLQ24C1401), and the National Natural Science Foundation of China (U23A6006, 32270146).

## Conflicts of Interest

The authors declare no conflicts of interest.

## Supporting information


**Figure S1:** Comparative analysis of *Toll7* transcriptional levels in non‐viruliferous and viruliferous third‐instar *Laodelphax striatellus* nymphs. The transcriptional profile of *Toll7* was quantitatively assessed across independent biological replicates, with 20–23 individuals sampled per group. Intergroup comparisons were performed using *t*‐test method, with significant differential expression (*p* < 0.01) denoted by double asterisks (**). Error bars depict the standard error (SE) of the mean.

**Figure S2:** Phylogenetic analysis of Toll7 and interactions between Toll7 and RSV‐encoded proteins. (A) Phylogenetic relationships of Toll7 among diverse arthropods and mammals. The phylogenetic tree was reconstructed using the maximum‐likelihood method implemented in MEGA 7.0 software. The Toll7 homolog from *Laodelphax striatellus* is highlighted in bold black font. (B) Yeast two‐hybrid assay demonstrating interactions between Toll7 and RSV‐encoded proteins (RSV‐NSvc2‐N, RSV‐MP, RSV‐NS3 and RSV‐NS4). Yeast cells co‐transformed with the indicated plasmid combinations were initially grown on synthetic dropout medium SD/−Leu/−Trp to confirm transformation. Protein–protein interactions were subsequently assessed on selective medium SD/−Ade/−His/−Leu/−Trp. Images were captured following a 3‐day incubation period at 30°C.

**Figure S3:** Interactions between mTOR and RSV‐encoded proteins. (A) Conserved domain analysis of mTOR, highlighting the presence of the TEL domain. (B) Yeast two‐hybrid assay to investigate interactions between the mTOR‐TEL domain and RSV‐encoded proteins (RSV‐*NSvc2‐C*, RSV‐*NP*, RSV‐*NS3*, RSV‐*NS4* and RSV‐*MP*). Yeast cells co‐transformed with the indicated plasmid combinations were initially selected on synthetic dropout medium SD/−Leu/−Trp. Protein–protein interactions were subsequently assessed on selective medium SD/−Ade/−His/−Leu/−Trp. The positive control group (AD‐T7‐t and BD‐53) was included to validate the assay. Images were captured after a 3‐day incubation period at 30°C.

**Figure S4:** Effect of *Toll7* silencing followed by virus acquisition on ATG8‐I and ATG8‐II protein in non‐viruliferous *Laodelphax striatellus* .

**Table S1:** Primers used in this study.

## Data Availability

The data that support the findings of this study are available from the corresponding author upon reasonable request.

## References

[mpp70259-bib-0001] Alessi, D. R. , M. Andjelkovic , B. Caudwell , et al. 1996. “Mechanism of Activation of Protein Kinase B by Insulin and IGF‐1.” EMBO Journal 15: 6541–6551. 8978681 PMC452479

[mpp70259-bib-0002] Barton, G. M. 2007. “Viral Recognition by Toll‐Like Receptors.” Seminars in Immunology 19: 33–40. 17336545 10.1016/j.smim.2007.01.003

[mpp70259-bib-0003] Bayascas, J. R. , and D. R. Alessi . 2005. “Regulation of Akt/PKB Ser473 Phosphorylation.” Molecular Cell 18: 143–145. 15837416 10.1016/j.molcel.2005.03.020

[mpp70259-bib-0004] Belvin, M. P. , and K. V. Anderson . 1996. “A conserved Signaling Pathway: The Drosophila Toll‐Dorsal Pathway.” Annual Review of Cell and Developmental Biology 12: 393–416. 10.1146/annurev.cellbio.12.1.393 8970732

[mpp70259-bib-0005] Chang, H. , X. Li , Q. Cai , et al. 2017. “The PI3K/Akt/mTOR Pathway is Involved in CVB3‐Induced Autophagy of HeLa Cells.” International Journal of Molecular Medicine 40: 182–192. 28560385 10.3892/ijmm.2017.3008 PMC5466389

[mpp70259-bib-0006] Chen, X. , J. Yu , W. Wang , et al. 2020. “A Plant Virus Ensures Viral Stability in the Hemolymph of Vector Insects through Suppressing Prophenoloxidase Activation.” mBio 11: e01453‐20. 32817105 10.1128/mBio.01453-20 PMC7439478

[mpp70259-bib-0007] Cherry, S. 2009. “VSV Infection is Sensed by *Drosophila*, Attenuates Nutrient Signaling, and Thereby Activates Antiviral Autophagy.” Autophagy 5: 1062–1063. 19713743 10.4161/auto.5.7.9730

[mpp70259-bib-0008] Cho, D. , J. W. Mier , and M. B. Atkins . 2009. “PI3K/Akt/mTOR Pathway: A Growth and Proliferation Pathway.” In Renal Cell Carcinoma: Molecular Targets and Clinical Applications, edited by R. M. Bukowski , R. A. Figlin , and R. J. Motzer , 267–285. Springer Nature.

[mpp70259-bib-0009] Czech, M. P. 2000. “PIP2 and PIP3: Complex Roles at the Cell Surface.” Cell 100: 603–606. 10761925 10.1016/s0092-8674(00)80696-0

[mpp70259-bib-0010] Datta, S. R. , A. Brunet , and M. E. Greenberg . 1999. “Cellular Survival: A Play in Three Akts.” Genes & Development 13: 2905–2927. 10579998 10.1101/gad.13.22.2905

[mpp70259-bib-0011] Delgado, M. , and V. Deretic . 2009. “Toll‐Like Receptors in Control of Immunological Autophagy.” Cell Death and Differentiation 16: 976–983. 19444282 10.1038/cdd.2009.40 PMC2788936

[mpp70259-bib-0012] Ersahin, T. , N. Tuncbag , and R. Cetin‐Atalay . 2015. “The PI3K/AKT/mTOR Interactive Pathway.” Molecular BioSystems 11: 1946–1954. 25924008 10.1039/c5mb00101c

[mpp70259-bib-0013] Falk, B. W. , and J. H. Tsai . 1998. “Biology and Molecular Biology of Viruses in the Genus *Tenuivirus* .” Annual Review of Phytopathology 36: 139–163. 10.1146/annurev.phyto.36.1.139 15012496

[mpp70259-bib-0014] Ferreira, A. G. , H. Naylor , S. S. Esteves , I. S. Pais , N. E. Martins , and L. Teixeira . 2014. “The Toll‐Dorsal Pathway is Required for Resistance to Viral Oral Infection in *Drosophila* .” PLoS Pathogens 10: e1004507. 25473839 10.1371/journal.ppat.1004507 PMC4256459

[mpp70259-bib-0015] Hart, J. R. , and P. K. Vogt . 2011. “Phosphorylation of AKT: A Mutational Analysis.” Oncotarget 2: 467–476. 21670491 10.18632/oncotarget.293 PMC3139455

[mpp70259-bib-0016] Hayano, Y. , T. Kakutani , T. Hayashi , and Y. Minobe . 1990. “Coding Strategy of Rice Stripe Virus: Major Nonstructural Protein is Encoded in Viral RNA Segment 4 and Coat Protein in RNA Complementary to Segment 3.” Virology 177: 372–374. 2141205 10.1016/0042-6822(90)90493-b

[mpp70259-bib-0017] He, Y.‐J. , G. Lu , Y.‐H. Qi , et al. 2021. “Activation of Toll Immune Pathway in an Insect Vector Induced by a Plant Virus.” Frontiers in Immunology 11: 613957. 33488623 10.3389/fimmu.2020.613957 PMC7821435

[mpp70259-bib-0018] He, Y.‐J. , G. Lu , B.‐J. Xu , et al. 2024. “Maintenance of Persistent Transmission of a Plant Arbovirus in Its Insect Vector Mediated by the Toll‐Dorsal Immune Pathway.” Proceedings of the National Academy of Sciences of the United States of America 121: e2315982121. 38536757 10.1073/pnas.2315982121 PMC10998634

[mpp70259-bib-0019] Hemmi, H. , O. Takeuchi , T. Kawai , et al. 2000. “A Toll‐Like Receptor Recognizes Bacterial DNA.” Nature 408: 740–745. 11130078 10.1038/35047123

[mpp70259-bib-0020] Heras‐Sandoval, D. , J. M. Pérez‐Rojas , J. Hernández‐Damián , and J. Pedraza‐Chaverri . 2014. “The Role of PI3K/AKT/mTOR Pathway in the Modulation of Autophagy and the Clearance of Protein Aggregates in Neurodegeneration.” Cellular Signalling 26: 2694–2701. 25173700 10.1016/j.cellsig.2014.08.019

[mpp70259-bib-0021] Hogenhout, S. A. , E.‐D. Ammar , A. E. Whitfield , and M. G. Redinbaugh . 2008. “Insect Vector Interactions With Persistently Transmitted Viruses.” Annual Review of Phytopathology 46: 327–359. 10.1146/annurev.phyto.022508.092135 18680428

[mpp70259-bib-0022] Horng, T. , and R. Medzhitov . 2001. “ *Drosophila* MyD88 is an Adapter in the Toll Signaling Pathway.” Proceedings of the National Academy of Sciences of the United States of America 98: 12654–12658. 11606776 10.1073/pnas.231471798 PMC60109

[mpp70259-bib-0023] Hu, X. , Y. Yagi , T. Tanji , S. Zhou , and Y. T. Ip . 2004. “Multimerization and Interaction of Toll and Spätzle in *Drosophila* .” Proceedings of the National Academy of Sciences of the United States of America 101: 9369–9374. 15197269 10.1073/pnas.0307062101 PMC438983

[mpp70259-bib-0024] Janeway, C. A., Jr. , and R. Medzhitov . 2002. “Innate Immune Recognition.” Annual Review of Immunology 20: 197–216. 10.1146/annurev.immunol.20.083001.084359 11861602

[mpp70259-bib-0025] Jia, D. , G. Luo , H. Guan , et al. 2024. “Arboviruses Antagonize Insect Toll Antiviral Immune Signaling to Facilitate the Coexistence of Viruses With Their Vectors.” PLoS Pathogens 20: e1012318. 38865374 10.1371/journal.ppat.1012318 PMC11198909

[mpp70259-bib-0026] Jung, C. H. , S.‐H. Ro , J. Cao , N. M. Otto , and D. H. Kim . 2010. “mTOR Regulation of Autophagy.” FEBS Letters 584: 1287–1295. 20083114 10.1016/j.febslet.2010.01.017 PMC2846630

[mpp70259-bib-0027] Leulier, F. O. , and B. Lemaitre . 2008. “Toll‐Like Receptors—Taking an Evolutionary Approach.” Nature Reviews Genetics 9: 165. 10.1038/nrg2303 18227810

[mpp70259-bib-0028] Li, H. , B. Yin , S. Wang , et al. 2018. “RNAi Screening Identifies a New Toll From Shrimp *Litopenaeus vannamei* That Restricts WSSV Infection Through Activating Dorsal to Induce Antimicrobial Peptides.” PLoS Pathogens 14: e1007109. 30256850 10.1371/journal.ppat.1007109 PMC6175524

[mpp70259-bib-0029] Li, J. , I. B. Andika , J. Shen , et al. 2013. “Characterization of Rice Black‐Streaked Dwarf Virus‐ and Rice Stripe Virus‐Derived siRNAs in Singly and Doubly Infected Insect Vector *Laodelphax striatellus* .” PLoS One 8: e66007. 23776591 10.1371/journal.pone.0066007 PMC3679040

[mpp70259-bib-0030] Lin, H. , B. Li , M. Liu , H. Zhou , K. He , and H. Fan . 2020. “Nonstructural Protein 6 of Porcine Epidemic Diarrhea Virus Induces Autophagy to Promote Viral Replication via the PI3K/Akt/mTOR axis.” Veterinary Microbiology 244: 108684. 32402351 10.1016/j.vetmic.2020.108684 PMC7165116

[mpp70259-bib-0031] Lund, J. M. , L. Alexopoulou , A. Sato , et al. 2004. “Recognition of Single‐Stranded RNA Viruses by Toll‐Like Receptor 7.” Proceedings of the National Academy of Sciences of the United States of America 101: 5598–5603. 15034168 10.1073/pnas.0400937101 PMC397437

[mpp70259-bib-0032] Manning, B. D. , and L. C. Cantley . 2007. “AKT/PKB Signaling: Navigating Downstream.” Cell 129: 1261–1274. 17604717 10.1016/j.cell.2007.06.009 PMC2756685

[mpp70259-bib-0033] Medzhitov, R. 2001. “Toll‐Like Receptors and Innate Immunity.” Nature Reviews Immunology 1: 135–145. 10.1038/35100529 11905821

[mpp70259-bib-0034] Moy, R. H. , B. Gold , J. M. Molleston , et al. 2014. “Antiviral Autophagy Restricts Rift Valley Fever Virus Infection and is Conserved From Flies to Mammals.” Immunity 40: 51–65. 24374193 10.1016/j.immuni.2013.10.020 PMC3951734

[mpp70259-bib-0035] Nakamoto, M. , R. H. Moy , J. Xu , et al. 2012. “Virus Recognition by Toll‐7 Activates Antiviral Autophagy in *Drosophila* .” Immunity 36: 658–667. 22464169 10.1016/j.immuni.2012.03.003 PMC3334418

[mpp70259-bib-0036] Saxton, R. A. , and D. M. Sabatini . 2017. “mTOR Signaling in Growth, Metabolism, and Disease.” Cell 168: 960–976. 28283069 10.1016/j.cell.2017.02.004 PMC5394987

[mpp70259-bib-0037] Sun, H. , B. N. Bristow , G. Qu , and S. A. Wasserman . 2002. “A Heterotrimeric Death Domain Complex in Toll Signaling.” Proceedings of the National Academy of Sciences of the United States of America 99: 12871–12876. 12351681 10.1073/pnas.202396399 PMC130552

[mpp70259-bib-0038] Takahashi, M. a. , S. h. Toriyama , C. h. Hamamatsu , et al. 1993. “Nucleotide Sequence and Possible Ambisense Coding Strategy of Rice Stripe Virus RNA Segment 2.” Journal of General Virology 74: 769–773. 8468559 10.1099/0022-1317-74-4-769

[mpp70259-bib-0039] Takeda, K. , and S. Akira . 2003. “Toll Receptors and Pathogen Resistance.” Cellular Microbiology 5: 143–153. 12614458 10.1046/j.1462-5822.2003.00264.x

[mpp70259-bib-0040] Toriyama, S. 1986. “Rice Stripe Virus: Prototype of a New Group of Viruses That Replicate in Plants and Insects.” Microbiological Sciences 3: 347–351. 2856619

[mpp70259-bib-0041] Toriyama, S. , M. Takahashi , Y. Sano , T. Shimizu , and A. Ishihama . 1994. “Nucleotide Sequence of RNA 1, the Largest Genomic Segment of Rice Stripe Virus, the Prototype of the Tenuiviruses.” Journal of General Virology 75: 3569–3579. 7996149 10.1099/0022-1317-75-12-3569

[mpp70259-bib-0042] Towb, P. , A. Bergmann , and S. A. Wasserman . 2001. “The Protein Kinase Pelle Mediates Feedback Regulation in the *Drosophila* Toll Signaling Pathway.” Development 128: 4729–4736. 11731453 10.1242/dev.128.23.4729

[mpp70259-bib-0043] Valanne, S. , J.‐H. Wang , and M. Rämet . 2011. “The *Drosophila* Toll Signaling Pathway.” Journal of Immunology 186: 649–656. 10.4049/jimmunol.1002302 21209287

[mpp70259-bib-0044] Wang, W. , W. Zhao , J. Li , L. Luo , L. Kang , and F. Cui . 2017. “The c‐Jun N‐Terminal Kinase Pathway of a Vector Insect is Activated by Virus Capsid Protein and Promotes Viral Replication.” eLife 6: e26591. 28716183 10.7554/eLife.26591 PMC5515582

[mpp70259-bib-0045] Wang, Y. , and H. Zhang . 2019. “Regulation of Autophagy by mTOR Signaling Pathway.” In Autophagy: Biology and Diseases: Basic Science, 67–83. Springer Singapore. 10.1007/978-981-15-0602-4_3 31776980

[mpp70259-bib-0046] Weber, A. N. , S. Tauszig‐Delamasure , J. A. Hoffmann , et al. 2003. “Binding of the *Drosophila* Cytokine Spätzle to Toll is Direct and Establishes Signaling.” Nature Immunology 4: 794–800. 12872120 10.1038/ni955

[mpp70259-bib-0047] Werling, D. , and T. W. Jungi . 2003. “Toll‐Like Receptors Linking Innate and Adaptive Immune Response.” Veterinary Immunology and Immunopathology 91: 1–12. 12507844 10.1016/s0165-2427(02)00228-3

[mpp70259-bib-0048] Wu, G. , J. Wang , Y. Yang , et al. 2014. “Transgenic Rice Expressing Rice Stripe Virus NS3 Protein, a Suppressor of RNA Silencing, Shows Resistance to Rice Blast Disease.” Virus Genes 48: 566–569. 24557730 10.1007/s11262-014-1051-2

[mpp70259-bib-0049] Wu, Y.‐T. , H.‐L. Tan , Q. Huang , C. N. Ong , and H. M. Shen . 2009. “Activation of the PI3K‐Akt‐mTOR signaling pathway promotes necrotic cell death via suppression of autophagy.” Autophagy 5: 824–834. 19556857 10.4161/auto.9099

[mpp70259-bib-0050] Xi, J.‐C. , H.‐Y. Zang , L.‐X. Guo , et al. 2015. “The PI3K/AKT cell signaling pathway is involved in regulation of osteoporosis.” Journal of Receptor and Signal Transduction Research 35: 640–645. 26390889 10.3109/10799893.2015.1041647

[mpp70259-bib-0051] Xi, Z. , J. L. Ramirez , and G. Dimopoulos . 2008. “The *Aedes aegypti* toll pathway controls dengue virus infection.” PLoS Pathogens 4: e1000098. 18604274 10.1371/journal.ppat.1000098 PMC2435278

[mpp70259-bib-0052] Xiao, L. , L. L. Huang , H. M. He , F. S. Xue , and J. J. Tang . 2023. “Life history responses of the small brown planthopper *Laodelphax striatellus* to temperature change.” Journal of Thermal Biology 115: 8. 10.1016/j.jtherbio.2023.103626 37364441

[mpp70259-bib-0053] Xu, H.‐J. , J. Xue , B. Lu , et al. 2015. “Two insulin receptors determine alternative wing morphs in planthoppers.” Nature 519: 464–467. 25799997 10.1038/nature14286

[mpp70259-bib-0054] Xu, Y. , S. Fu , X. Tao , and X. Zhou . 2021. “Rice stripe virus: Exploring molecular weapons in the arsenal of a negative‐sense RNA virus.” Annual Review of Phytopathology 59: 351–371. 10.1146/annurev-phyto-020620-113020 34077238

[mpp70259-bib-0055] Xu, Z. , X. Han , D. Ou , et al. 2020. “Targeting PI3K/AKT/mTOR‐Mediated Autophagy for Tumor Therapy.” Applied Microbiology and Biotechnology 104: 575–587. 31832711 10.1007/s00253-019-10257-8

[mpp70259-bib-0056] Yao, M. , X. Liu , S. Li , et al. 2014. “Rice Stripe Tenuivirus NSvc2 Glycoproteins Targeted to the Golgi Body by the N‐Terminal Transmembrane Domain and Adjacent Cytosolic 24 Amino Acids via the COP I‐and COP II‐Dependent Secretion Pathway.” Journal of Virology 88: 3223–3234. 24390331 10.1128/JVI.03006-13 PMC3957912

[mpp70259-bib-0057] Yao, R. , and G. M. Cooper . 1995. “Requirement for Phosphatidylinositol‐3 Kinase in the Prevention of Apoptosis by Nerve Growth Factor.” Science 267: 2003–2006. 7701324 10.1126/science.7701324

[mpp70259-bib-0058] Yu, Y. L. , M. T. Zhang , Y. Huo , et al. 2021. “ *Laodelphax striatellus* Atg8 Facilitates Rice Stripe Virus Infection in an Autophagy‐Independent Manner.” Insect Science 28: 315–329. 32108430 10.1111/1744-7917.12771

[mpp70259-bib-0059] Zambon, R. A. , M. Nandakumar , V. N. Vakharia , and L. P. Wu . 2005. “The Toll Pathway is Important for an Antiviral Response in *Drosophila* .” Proceedings of the National Academy of Sciences of the United States of America 102: 7257–7262. 15878994 10.1073/pnas.0409181102 PMC1129099

[mpp70259-bib-0060] Zhang, Y. , B.‐X. Li , Q.‐Z. Mao , et al. 2023. “The JAK‐STAT Pathway Promotes Persistent Viral Infection by Activating Apoptosis in Insect Vectors.” PLoS Pathogens 19: e1011266. 36928081 10.1371/journal.ppat.1011266 PMC10069781

[mpp70259-bib-0061] Zhao, W. , J. Zhu , H. Lu , et al. 2022. “The Nucleocapsid Protein of Rice Stripe Virus in Cell Nuclei of Vector Insect Regulates Viral Replication.” Protein & Cell 13: 360–378. 33675514 10.1007/s13238-021-00822-1 PMC7936609

[mpp70259-bib-0062] Zheng, L. , Z. Du , C. Lin , et al. 2015. “Rice Stripe *Tenuivirus* p2 May Recruit or Manipulate Nucleolar Functions Through an Interaction With Fibrillarin to Promote Virus Systemic Movement.” Molecular Plant Pathology 16: 921–930. 25431002 10.1111/mpp.12220 PMC6638460

