# Supplementary figures and images for: A Plant Virus Glycoprotein Induces Autophagy by Activating the Toll7 Immune Pathway in Its Insect Vector
Source: Mol Plant Pathol. 2026 Apr 12;27(4):e70259. doi: 10.1111/mpp.70259 (PMC13071176; doi:10.1111/mpp.70259)

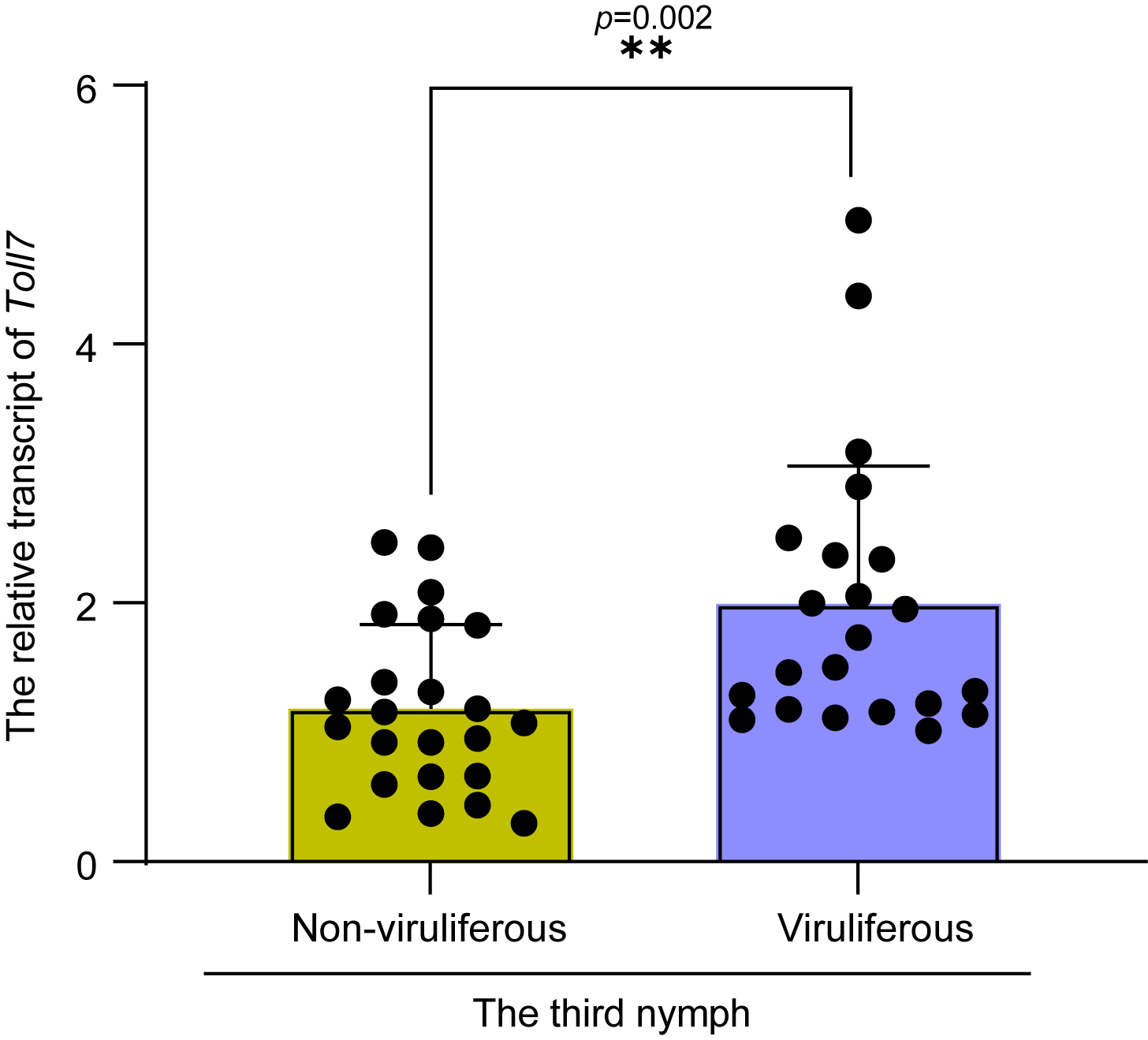

Supplement: Supplementary file 1 — Figure S1: Comparative analysis of Toll7 transcriptional levels in non‐viruliferous and viruliferous third‐instar Laodelphax striatellus nymphs. The transcriptional profile of Toll7 was quantitatively assessed across independent biological replicates, with 20–23 individuals sampled per group. Intergroup comparisons were performed using t‐test method, with significant differential expression (p < 0.01) denoted by double asterisks (**). Error bars depict the standard error (SE) of the mean. [file MPP-27-e70259-s002.tif]

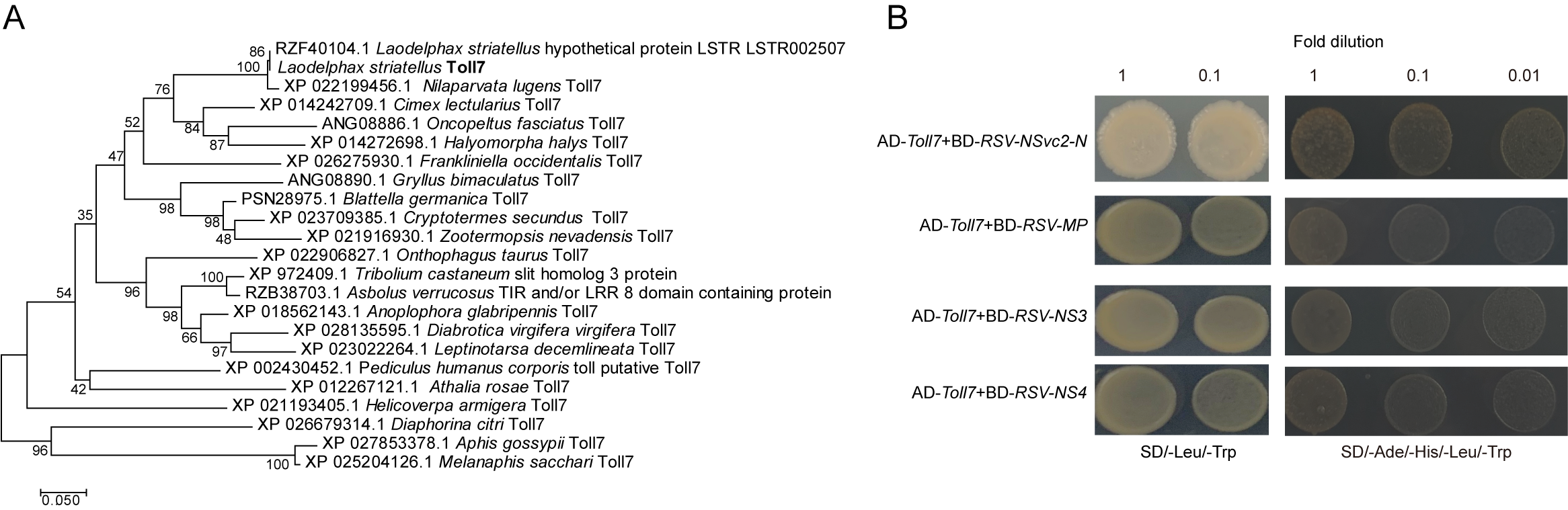

Supplement: Supplementary file 2 — Figure S2: Phylogenetic analysis of Toll7 and interactions between Toll7 and RSV‐encoded proteins. (A) Phylogenetic relationships of Toll7 among diverse arthropods and mammals. The phylogenetic tree was reconstructed using the maximum‐likelihood method implemented in MEGA 7.0 software. The Toll7 homolog from Laodelphax striatellus is highlighted in bold black font. (B) Yeast two‐hybrid assay demonstrating interactions between Toll7 and RSV‐encoded proteins (RSV‐NSvc2‐N, RSV‐MP, RSV‐NS3 and RSV‐NS4). Yeast cells co‐transformed with the indicated plasmid combinations were initially grown on synthetic dropout medium SD/−Leu/−Trp to confirm transformation. Protein–protein interactions were subsequently assessed on selective medium SD/−Ade/−His/−Leu/−Trp. Images were captured following a 3‐day incubation period at 30°C. [file MPP-27-e70259-s004.tif]

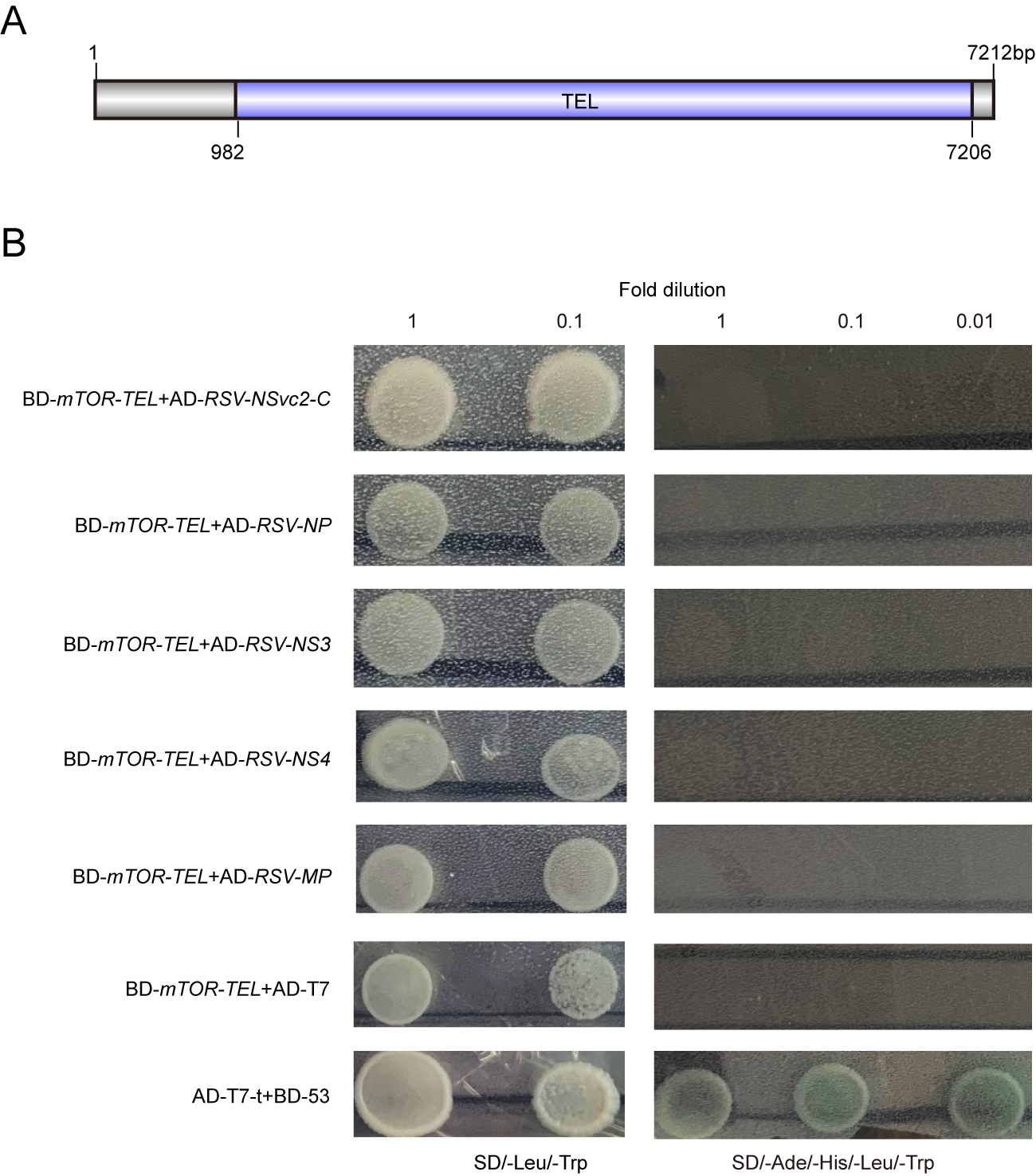

Supplement: Supplementary file 3 — Figure S3: Interactions between mTOR and RSV‐encoded proteins. (A) Conserved domain analysis of mTOR, highlighting the presence of the TEL domain. (B) Yeast two‐hybrid assay to investigate interactions between the mTOR‐TEL domain and RSV‐encoded proteins (RSV‐NSvc2‐C, RSV‐NP, RSV‐NS3, RSV‐NS4 and RSV‐MP). Yeast cells co‐transformed with the indicated plasmid combinations were initially selected on synthetic dropout medium SD/−Leu/−Trp. Protein–protein interactions were subsequently assessed on selective medium SD/−Ade/−His/−Leu/−Trp. The positive control group (AD‐T7‐t and BD‐53) was included to validate the assay. Images were captured after a 3‐day incubation period at 30°C. [file MPP-27-e70259-s005.tif]

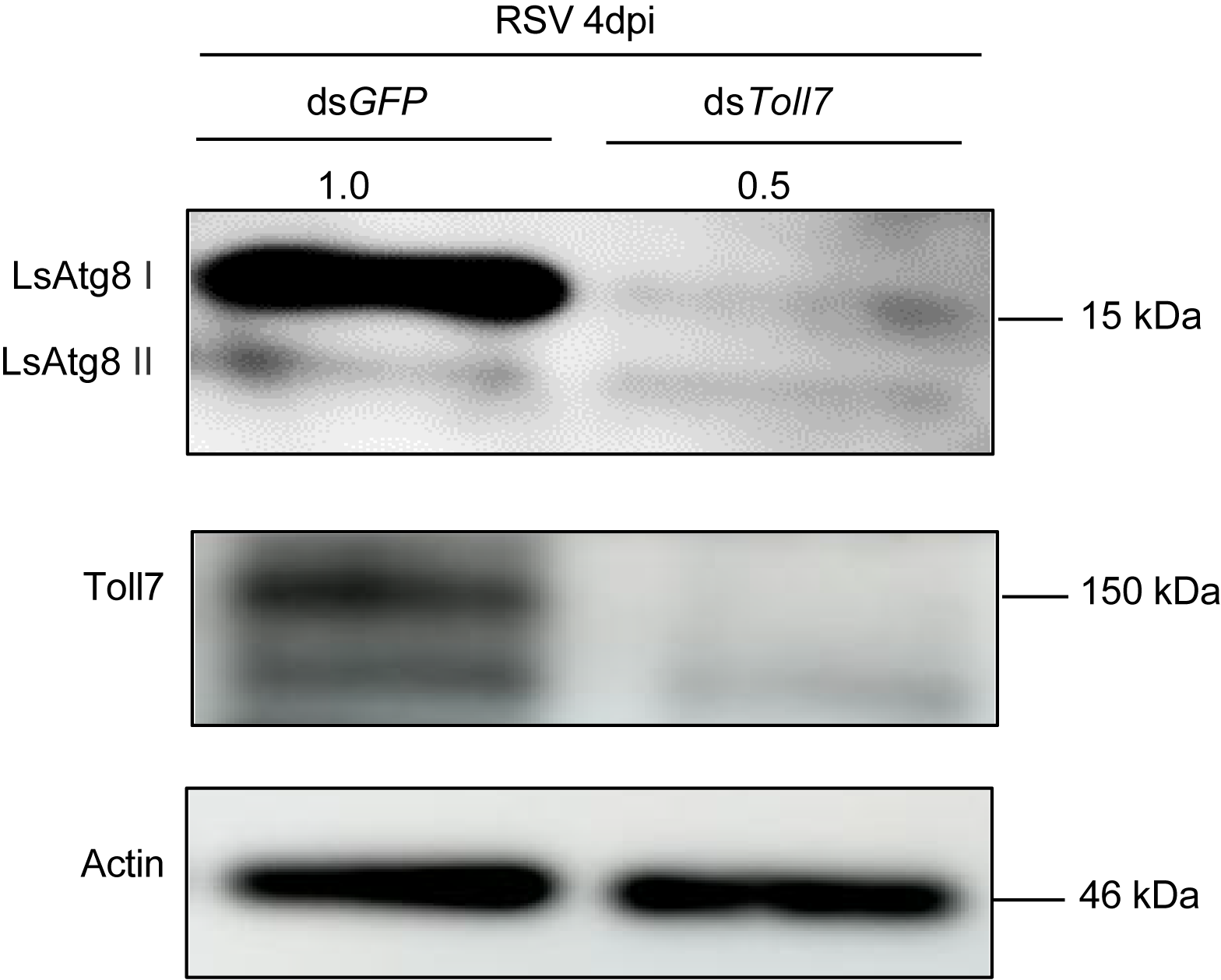

Supplement: Supplementary file 4 — Figure S4: Effect of Toll7 silencing followed by virus acquisition on ATG8‐I and ATG8‐II protein in non‐viruliferous Laodelphax striatellus . [file MPP-27-e70259-s003.tif]
