# Supplementary material for: A Plant Virus Glycoprotein Induces Autophagy by Activating the Toll7 Immune Pathway in Its Insect Vector
Source: Mol Plant Pathol. 2026 Apr 12;27(4):e70259. doi: 10.1111/mpp.70259 (PMC13071176; doi:10.1111/mpp.70259)
Supplement: Supplementary file 5 — Table S1: Primers used in this study. [file MPP-27-e70259-s001.docx]

Table S1 The primers used in this study.

| **Primer Name** | **Sequences（5’-3’）** |
| --- | --- |
| **For Yeast Two-Hybrid (Y2H) Assays** |  |
| pGAD-T7-*Toll7*-F | GACGTACCAGATTACGCTCATATGGCTCTTCTGCGGGTAT |
| pGAD-T7-*Toll7*-R | GCAGCTCGAGCTCGATGGATCCTCAGACTAGGTATGCCTGGA |
| pGAD-T7-*Toll7*-*TIR*-F | GACGTACCAGATTACGCTCATATGTACGATGGGTATGTTGTGTATA |
| pGAD-T7-*Toll7*-*TIR*-R | GCAGCTCGAGCTCGATGGATCCtta ACTCGGCATCACATATCGCAGT |
| pGBD-T7-*mTOR*-*TEL*-F | ATCTCAGAGGAGGACCTGCATATGTGCTACTCCGTGCTGAACCAGCGTTC |
| pGBD-T7-*mTOR*-*TEL*-R | GCCGCTGCAGGTCGACGGATCCTTACCAGAATGGGCATCTCCTCCAG |
| **For BIFC Assays** |  |
| nYFP-*Toll7*-F | ATCGAGGACTCCGGAGTCGACATGGACGAGTGCCAATGGACGT |
| nYFP-*Toll7*-R | GATCGGGGAAATTCGAGCTCTCAGACTAGGTATGCCTGGA |
| cYFP-*RSV-NSvc2-C* -F | CTGTACAAGTCCGGAGTCGACATGgaatcctgtcaagaccttgt |
| cYFP-*RSV-NSvc2-C* -R | GATCGGGGAAATTCGAGCTCttactttgggttgtccttgacta |
| **For DsRNA Synthesis** |  |
| ds-*Toll7*-F | TAATACGACTCACTATAGGGAGATCTTTCTCGCAACGACCTCC |
| ds-*Toll7*-R | TAATACGACTCACTATAGGGAGACGGCAAGATTGAGTGTGTGC |
| ds-*PI3K*-F | TAATACGACTCACTATAGGGAGATTGAGCGTGAGATGCAAGGT |
| ds-*PI3K* -R | TAATACGACTCACTATAGGGAGATCGTGGTCAGTGAGTCGTTG |
| ds-*AKT*-F | TAATACGACTCACTATAGGGAGATTCATTCCAGACAGCCGACC |
| ds-*AKT*-R | TAATACGACTCACTATAGGGAGAGCTCTTCGCTTCGGTCGATA |
| ds-*mTOR*-F | TAATACGACTCACTATAGGGAGA AGAGTTATCAGCGCGCCTAC |
| ds-*mTOR-*R | TAATACGACTCACTATAGGGAGA GCCACTGGCCAAGTTTCAAG |
| **For qRT-PCR** |  |
| q-*Toll7*-F | CATTGGGCAACAACCGATGG |
| q-*Toll7*-R | GGTGTTCTCGTTTCGTTCGC |
| q-*PI3K* -F | AAACTAAGCGTGGCTACGGA |
| q-*PI3K* -R | GACCAATGGGACGGTAGTGG |
| q-*AKT*-F | CGCTTATCGGGTTCAAAGCG |
| q-*AKT*-R | TGTCGGACTCGACGTGAAAG |
| q-*mTOR*-F | GCCGCCTATTGTCAGGCTAT |
| q-*mTOR*-R | CTCTGCTCACAAGAGGCACA |
| q-*Atg3*-F | TCACGTGGATCCCACAACTG |
| q-*Atg3*-R | TCCTCGTCTAGAAGGCCACT |
| q-*Atg5*-F | GACATTGAGCTACCCTGGGC |
| q-*Atg5*-R | CCATCTCCATCAGCTTGCGA |
| q-*Atg8*-F | CGTTATTCCCCCTACCAGCG |
| q-*Atg8*-R | TTTCCATAGACGTTCTCATCACT |
| q-*Atg9*-F | TGCACTACATACCGGACAGC |
| q-*Atg9*-R | CCATCTGTGCAAACGAGCAC |
| q-*Torc1*-F | ACCCGACCGCTATCATCAAC |
| q-*Torc1*-R | ACCAACAATCACCTCGGCT |
| q-*ULK1*-F | AGAGCACAACGAGACACTGG |
| q-*ULK1*-R | TGACCACGTTCTTGACGGAG |
| q-*Sqstm1*-F | GGCCGACAACTACAAGGTGA |
| q-*Sqstm1*-R | GGATGCGACGTCGGTATGAT |
| q-*LsActin*-F | AGTGCCCATCTACGAAGGTTACG |
| q-*LsActin*-R | CGGCGGTGGTGGTGAAGC |
| q-RSV-*NP*-F | ACCACAATCTTCGATGCGCT |
| q-RSV-*NP*-R | CCAACCCGCTGACTTCAGAT |
| **For Virus Detection** |  |
| RSV-*NP*-F | TGCCTCATCCTCGAAGAACTCC |
| RSV-*NP*-R | AGCCAGCCACTCTAGCTGATTT |
| **For Protein Purification** |  |
| Pet-28a-*Toll7*-F | CTGGTGCCGCGCGGCAGCCATATGGACGAGTGCCAATGGACGTCC |
| Pet-28a-*Toll7*-R | ACGGAGCTCGAATTCGAATCCTCAGCCTTCATCTACCTGGTTGCT |
|  |  |
|  |  |
|  |  |
